# Molecular Composition and Ultrastructure of the Caveolar Coat Complex

**DOI:** 10.1371/journal.pbio.1001640

**Published:** 2013-08-27

**Authors:** Alexander Ludwig, Gillian Howard, Carolina Mendoza-Topaz, Thomas Deerinck, Mason Mackey, Sara Sandin, Mark H. Ellisman, Benjamin J. Nichols

**Affiliations:** 1 Medical Research Council Laboratory of Molecular Biology, Cambridge, United Kingdom; 2 National Center for Microscopy and Imaging Research, University of California San Diego, La Jolla, California, United States of America; 3 School of Biological Sciences, Nanyang Technical University, Singapore; Princeton University, United States of America

## Abstract

Caveolae are an abundant feature of the plasma membrane of many mammalian cell types, and have key roles in mechano-transduction, metabolic regulation, and vascular permeability. Caveolin and cavin proteins, as well as EHD2 and pacsin 2, are all present in caveolae. How these proteins assemble to form a protein interaction network for caveolar morphogenesis is not known. Using *in vivo* crosslinking, velocity gradient centrifugation, immuno-isolation, and tandem mass spectrometry, we determine that cavins and caveolins assemble into a homogenous 80S complex, which we term the caveolar coat complex. There are no further abundant components within this complex, and the complex excludes EHD2 and pacsin 2. Cavin 1 forms trimers and interacts with caveolin 1 with a molar ratio of about 1∶4. Cavins 2 and 3 compete for binding sites within the overall coat complex, and form distinct subcomplexes with cavin 1. The core interactions between caveolin 1 and cavin 1 are independent of cavin 2, cavin 3, and EHD2 expression, and the cavins themselves can still interact in the absence of caveolin 1. Using immuno-electron microscopy as well as a recently developed protein tag for electron microscopy (MiniSOG), we demonstrate that caveolar coat complexes form a distinct coat all around the caveolar bulb. In contrast, and consistent with our biochemical data, EHD2 defines a different domain at the caveolar neck. 3D electron tomograms of the caveolar coat, labeled using cavin-MiniSOG, show that the caveolar coat is composed of repeating units of a unitary caveolar coat complex.

## Abstract 2

The single protein caveolar coat complex comprises only cavins and caveolins, coats the caveolar bulb, and is probably responsible for creating caveolae.

## Author Summary

Caveolae are flask-shaped invaginations in the plasma membrane of many mammalian cell types, and are particularly abundant in fat cells, muscle cells, and the cells that line blood vessels. Although caveolae are likely to be important for cellular responses to mechanical stress, intracellular trafficking, and signaling events, we still lack an understanding of the precise molecular mechanisms for how they form and carry out these functions. Here we address the question of how caveolae are made. Recent years have seen a considerable expansion of the catalogue of known protein components present in caveolae. Our study shows that the main protein components, cavins and caveolins, assemble into one specific complex. We reveal how different amounts of two caveolar proteins, cavin 2 and cavin 3, may be incorporated into this single type of complex, thereby potentially conferring different functional properties on caveolae. Using electron microscopy, we demonstrate that the protein complex is distributed all around the membrane bulb of caveolae, and so can be truly described as the caveolar coat. The caveolar coat excludes the protein EHD2, which regulates the dynamics of caveolae—this protein has a distinct distribution at the caveolar neck. These findings provide the basis for a more complete understanding of the network of protein interactions that produces caveolae.

## Introduction

Caveolae, plasma membrane invaginations with a diameter of about 50–80 nm and a characteristic flask-like shape, were first identified 60 y ago by electron microscopy [1],[2]. They are found in many different cell types, and are particularly abundant in endothelial cells, adipocytes, and muscle cells [3]–[5]. Caveolin 1 is the major integral membrane protein in caveolae, and is essential for their formation in nonmuscle cells [6]–[8]. There are two further caveolin proteins. Caveolin 2 has the same distribution as caveolin 1, and hetero-oligomerises with this protein, but is not essential for forming caveolae [9],[10]. Caveolin 3 is only expressed in striated muscle and is required for making caveolae in this tissue [11]. Mice lacking *caveolin 1* have multiple phenotypes including hyperglycaemia, lipidosis, and changes in endothelial permeability [8],[12]–, and humans with a loss of function mutation in *caveolin 1* are severely lipodystrophic [16]. The molecular and cellular causes of these phenotypes are not completely understood, but caveolae have been proposed to act in a variety of ways, including as endocytic vesicles, as mechanoprotective or mechanosensing membrane reservoirs, as regulators of lipid transport, and as scaffolds for signaling events [3]–[5],[17],[18].

Caveolins were long believed to be the sole protein component of caveolae, and they clearly have a central role in biogenesis of these structures. Direct evidence for this is provided by experiments showing that expression of caveolin in bacteria is sufficient to generate caveolae-like membrane vesicles [19]. Recently, however, the list of caveolar components has been considerably expanded, with the identification of cavin proteins [5],[20]–[26], EHD2 [27]–[29], and pacsin 2 [29],[30]. This implies that caveolar biogenesis and function involves a complex set of proteins, but how these proteins assemble physically and spatially to generate caveolae has yet to be fully elucidated.

The cavins (cavin 1, 2, 3, and 4) localise to caveolae, and are important for their formation and dynamics [5],[20]–[26]. It should be noted that the cavin nomenclature is not the same as the standard gene names for this family (cavin 1, PTRF; cavin 2, SDPR; cavin 3, PRKCDBP; cavin 4, MURC; Cavin 3 is also frequently referred to as SRBC [5]). Cavin 1 is expressed in all cell types that express caveolins, and is essential for making caveolae *in vivo* [24],[31]. Phenotypes of *cavin 1* knockout mice resemble those of *caveolin 1 caveolin 3* double knockout mice, implying a central role for cavin 1 in caveolar biogenesis [14],[31],[32]. In contrast to cavin 1, expression of cavins 2, 3, and 4 is more cell and tissue-specific, with cavin 4 only being expressed in striated muscle [22],[33]. Intriguingly, cavin 2 is required for morphogenesis of caveolae in the endothelia of some tissues but not others, and cavin 3 appears to be dispensable for forming caveolae [34]. Cavins are present in large complexes that can be detected on sucrose gradients, and the apparent size of these complexes differs between tissues [34],[35]. Cavins can be co-immunoprecipitated with each other, and cavins 1 and 2 interact directly [21]. Overexpression of cavin 2 distorts and elongates caveolae, while cavin 3 depletion reduces intracellular transport of caveolar vesicles [20],[21]. These data suggest that *in vivo* caveolae may contain different complements of cavin proteins, and that cavins 2 and 3 may regulate caveolar function and dynamics in a cell-type-specific manner. How different complements of cavins can be incorporated into morphologically uniform caveolae remains unclear.

Caveolae contain an estimated 140–180 caveolin molecules [36] and oligomerisation of caveolins is likely to be critical for caveolae formation. Oligomerisation is cholesterol-dependent, and occurs initially in the trans-Golgi network, resulting in 8S complexes with an estimated 14–16 caveolin molecules [10],[35],[37]. Upon vesicular transport to the plasma membrane, such caveolin oligomers somehow assemble into higher order oligomeric complexes, which are likely to constitute a key structural unit of the caveolar coat [38]. Cavin proteins first co-localise with caveolins after delivery to the plasma membrane, but the nature of this association is not clear, as cavins and caveolins do not co-fractionate on sucrose gradients of detergent-solubilised cell lysates [35]. Moreover, whether the additional caveolar proteins EHD2 and pacsin 2, both of which are likely to regulate caveolar function or dynamics in some way [27]–[30], associate with caveolins and/or cavins directly or with other determinants within caveolar membranes is unknown.

Electron microscopy (EM) techniques have been used to try and ascertain whether there is a protein coat that surrounds caveolae, and to determine its organisation. Platinum and chromium coating of plasma membrane fragments suggests the presence of spiral ridges or striations on the bulb that can be detected by scanning EM [6],[39]. A similar distribution of densities is seen after high-pressure freezing and freeze-substitution [40], and in conventionally stained ultrathin sections periodic local maxima in electron density are observed around the caveolar membrane [41]. However, the organisation of such protein densities as well as the actual shape of caveolae is contingent on the fixation method used [42], and so the relationship between the striations observed by scanning EM and the protein densities seen by transmission EM is not clear. It has been suggested that the caveolar neck forms a separate domain distinct from the caveolar bulb, but neither the identity of the protein components around the bulb nor those around the neck are fully defined [41],[43]. Although some studies report that caveolins are found all around the caveolar bulb [44],[45], others report a more restricted distribution to the sides or neck of caveolae [46]. Inherent limitations of immuno-labeling, including the possibility that epitopes may not be equally accessible all over the caveolar surface and the reduced spatial resolution provided by combining primary and secondary antibodies, mean that it has been hard to address this issue unequivocally [43],[47]. Finally, the subcaveolar distribution of more recently identified components of caveolae, such as the cavins and EHD2, has yet to be addressed.

In the work reported here we have addressed fundamental questions central to an understanding of the protein machinery responsible for generating caveolae. We determine the identity and biochemical properties of the complexes into which caveolins and cavins assemble. We find that cavins and caveolins, but not EHD2 and pacsin 2, are found in a specific 80S complex, which we term the caveolar coat complex. Both immuno-EM and EM labeling with MiniSOG fusion proteins [48] show that this unitary complex does indeed localize all around the caveolar bulb. EHD2 defines a spatially and biochemically distinct domain at the neck of caveolae, and is not required for formation of the coat complex. These data provide conceptual advances in our understanding of how caveolae are generated.

## Results

### Caveolins and Cavins Assemble Into an 80S Caveolar Coat Complex

Caveolin 1 has been reported to exist as a labile high molecular weight complex of about 70S, as determined by velocity gradient centrifugation [35],[43]. Such oligomeric complexes of caveolin 1 are readily lost upon extraction of cells with detergents known to fully solubilize membranes rich in cholesterol and sphingolipids, and the apparent size of caveolin 1 complexes is highly sensitive to the nature of the detergent used for solubilisation (Figure S1) [35]. We looked for ways to stabilise complexes containing caveolin prior to cell lysis. These experiments were carried out in a clonal HeLa cell line stably expressing caveolin-1-GFP at about 20% of the level of endogenous caveolin 1 (Figure S2A).

**Figure 1:**
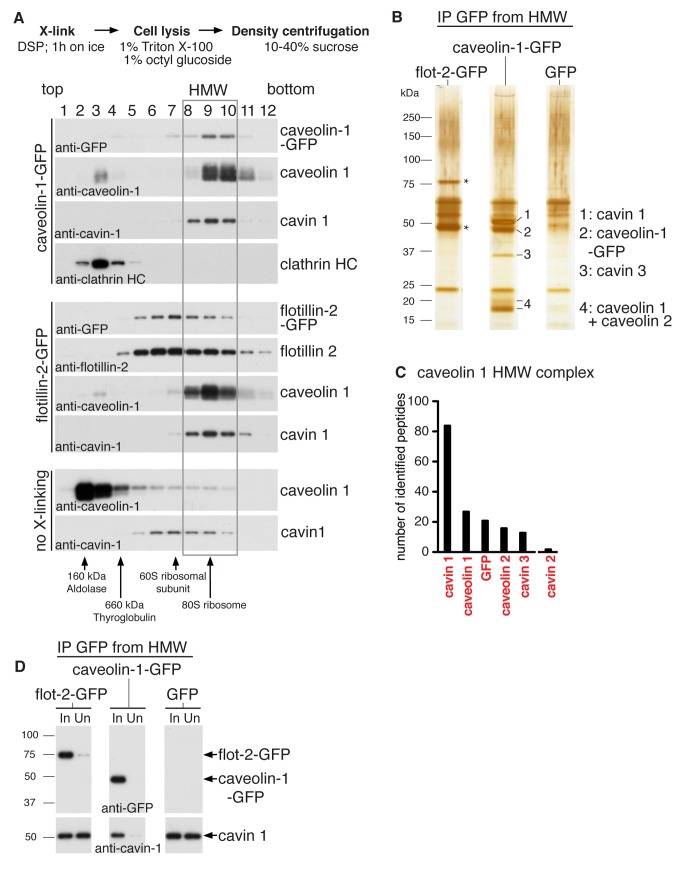
Caveolins and cavins assemble into an 80S complex. (A) HeLa cells were cross-linked with DSP and solubilised in 1% Triton X-100/1% octyl glucoside. Lysates were fractionated on 10–40% sucrose gradients, followed by Western blotting of gradient fractions 1–12 using antibodies against caveolin 1, cavin 1, GFP, clathrin heavy chain, or flotillin 2. Gradients were prepared from cells expressing caveolin-1-GFP (top panel) or flotillin-2-GFP as a control (middle panel). The bottom panel shows a gradient from flotillin-2-GFP expressing cells that had not been cross-linked prior to cell lysis. The high molecular weight (HMW) peak 8–10 of caveolin 1 and cavin 1 is boxed. The distribution of molecular weight protein standards in the gradients is indicated on the bottom. (B) Caveolin-1-GFP or flotillin-2-GFP (to provide a control) was immuno-isolated from pooled gradient fractions 8–10, indicated “HMW” in (A). A control immuno-isolation was also performed from fractions 8–10 of cells expressing GFP. Eluted proteins were visualized by silver staining. Cavin 1, caveolin-1-GFP, cavin 3, and caveolins are indicated. The bands specific to the flotillin-2-GFP immunoprecipitation, marked with asterisks (*) are flotillin-2-GFP, and endogenous flotillins 1 and 2. (C) The caveolin 1 HMW complex was analyzed by LC-MS/MS, and the graph shows the number of peptides for each protein identified. Proteins are ranked left to right by number of peptides identified. (D) Western blots of the starting material (In) and unbound material (Un) after immuno-isolation of caveolin-1-GFP, flotillin-2-GFP, or GFP from pooled HMW fractions 8–10. Membranes were probed with anti-GFP or anti-cavin 1 antibodies.

We found that cross-linking of live HeLa cells with the membrane permeable and reversible cross-linker DSP (dithiobis(succinimidylpropionate)) efficiently and reproducibly stabilised a high molecular weight complex containing caveolin 1 (Figure 1A). Upon cross-linking, caveolin 1 was found almost exclusively in a single sharp peak in fractions 8–10 of 10–40% sucrose velocity gradients (Figure 1A and Figure S1). This was the case even when cells were lysed in 2% w/v (70 mM) octyl-glucoside (OG), a condition where without cross-linker most caveolin 1 is found in the top four fractions of the gradient (Figure S1) [35].

In the presence of cross-linker, cavin 1 co-fractionated precisely with caveolin 1 (Figure 1A), whereas without cross-linking cavin 1 was found in a broad peak in the centre of the gradient (centred on fractions 6/7, about 60S; [35]) and did not co-fractionate with caveolin 1 (Figure 1A). Using the profile of cellular 80S ribosomes and purified 60S ribosomal subunits as a reference, we estimated that the cross-linked high molecular weight caveolin and cavin complexes have a sedimentation rate of about 80S (Figure S2B). 80S caveolin complexes were also detected in control cell lines expressing flotillin-2-GFP or GFP alone, and so were not dependent on the presence of caveolin-1-GFP (Figure 1A). Caveolin-1-GFP had the same distribution as endogenous caveolin 1 in the gradient (Figure 1A). Cross-linking did not change the distribution of caveolin 1 or cavin 1 when studied by immunofluorescence, and did not alter the appearance or brightness of either protein as they co-localised in puncta that are likely to correspond to individual caveolae (Figure S3A and S3B). Therefore, cross-linking with DSP does not itself induce redistribution of cavins or caveolin 1 within cells before lysis. These results suggest that cross-linking of live cells with DSP stabilises caveolin/cavin interactions that are otherwise lost during solubilisation with detergents, and thereby allows identification of a large 80S complex containing cavins and caveolins.

To determine the protein composition of the 80S complex, and to confirm that co-fractionation of caveolin 1 and cavin 1 after cross-linking indeed reflects co-assembly of both proteins into the same complex, caveolin-1-GFP was immuno-isolated from pooled fractions 8–10 (HMW, high molecular weight fractions) using magnetic anti-GFP beads (Figure 1B). Immuno-isolation from the same fractions of gradients of cell lysates from flotillin-2-GFP or GFP expressing cells served as controls. The complexes were washed extensively and eluted with a pH shift. Eluates were reduced with DTT to disassemble DSP cross-links, and analysed by SDS-PAGE and silver staining. This revealed the presence of four major bands specific to the caveolin-1-GFP immunoprecipitate (Figure 1B). Western blotting (Figure S1C), and excision of the relevant bands for analysis by mass spectrometry, both confirmed that these correspond to caveolin-1-GFP, cavin 1, cavin 3, and caveolins 1 and 2. Tandem mass spectrometry of the immuno-precipitated 80S complex identified all of the above proteins, and these were the only abundant proteins detected. Cavin 2 was also detected in the complex, though at significantly lower levels (Figure 1C, File S1). Western blotting of the isolated complex confirmed that all caveolar proteins co-purified specifically with caveolin-1-GFP, and not with affinity-purified flotillin-2-GFP complexes or mock purifications from GFP control cells (Figure S2C).

Cavin 1 yielded the most tryptic peptides identified by mass spectrometry analysis of isolated 80S complexes, and was the strongest band on silver-stained gels (Figure 1B and 1C, File S1). In addition, immuno-isolation of caveolin-1-GFP from the HMW fractions caused cavin 1 to be efficiently depleted from these fractions, showing that the large majority of cellular cavin 1 is present in complexes with caveolin 1 (Figure 1D). Together these data show that caveolins 1 and 2 and cavins 1, 2, and 3 assemble into an 80S complex, and that cavin 1 is a major component of this complex. Moreover, we can state that there are no further abundant protein components in the isolated complex. We hereafter refer to this complex as the caveolar coat complex.

### There Is a Single Species of Caveolar Coat Complex

To study the role of cavins 1, 2, and 3 in the assembly of the caveolar coat complex, we generated separate HeLa cell lines stably expressing each protein with a C-terminal TEV-GFP-10×His tag (from now on referred to as cavin-1, -2, or -3-GFP). All cavin fusion proteins localised to caveolae by light microscopy (Figure S4A, Table S1), and cavins 1, 2, and 3 co-localised extensively with each other (Figure S4B). Fusion proteins were expressed at low levels, with cavin-1-GFP being expressed at about 20% of endogenous cavin 1 (Figure S4C). Endogenous cavin 2 is difficult to detect in HeLa cells with available antibodies (although it is present, albeit at low levels, as it is detected by mass spectrometry, Figure 1E and File S1), so cavin-2-GFP is likely to be present at significantly higher levels than endogenous cavin 2 in the cavin-2-GFP cell line. The expression of endogenous cavin 3 was specifically down-regulated in several independent cavin-3-GFP-expressing clonal cell lines, resulting in cell lines expressing cavin-3-GFP instead of cavin 3. In the cell line used for the experiments presented here, expression of cavin-3-GFP was similar to that of cavin 3 in control cells (Figure S4C).

**Figure 2:**
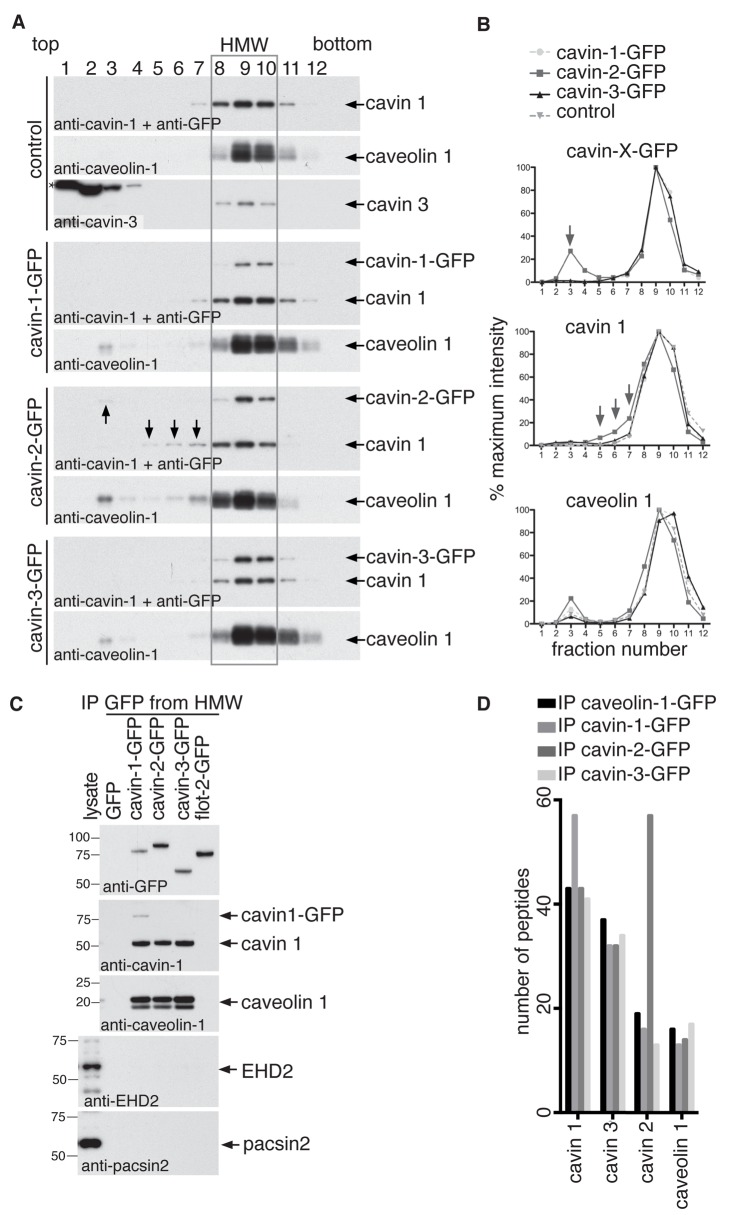
There is a single species of caveolar coat complex. (A) Cross-linked and detergent solubilised (1% Triton X-100/1% octyl glucoside) HeLa cell extracts were fractionated by velocity centrifugation (10–40% sucrose), followed by Western blotting of fractions 1–12. Gradients were prepared from cells expressing either GFP (the control cells), cavin 1-GFP, cavin 2-GFP, or cavin 3-GFP. Membranes were probed with antibodies against caveolin 1, cavin 1, cavin 3, or GFP. The high molecular weight (HMW) peak of caveolin 1 and cavins is boxed. The strong band in the cavin 3 blot (marked with an asterisk (*); fractions 1–4) are nonspecific: they are also observed in *cavin 3* knockout cells and in HeLa cells depleted of cavin 3 by siRNA (see Figure 5A). (B) Quantification of the distribution of cavin 1, 2, and-3-GFP, cavin 1, and caveolin 1 in velocity gradients as shown in (A). Relative protein amounts were determined by densitometry of Western blots. The data are expressed as an average percentage of protein in each fraction calculated from four independent experiments. Control was GFP-expressing cells. (C) Cavin 1, 2, and-3-GFP were immuno-isolated from pooled gradient HMW fractions 8–10. Control immuno-isolations were performed from pooled fractions 8–10 of cells expressing GFP or flotillin-2-GFP. Eluted proteins were analysed by Western blotting using antibodies against GFP, cavin 1, caveolin 1, EHD2, and pacsin 2. Note the absence of EHD2 and pacsin 2 from the caveolar coat complex. (D) HMW complexes immuno-isolated from cross-linked HeLa cells expressing caveolin-1-GFP, or cavin 1, 2, or-3-GFP were analyzed by LC-MS/MS. The graph is showing the number of peptides identified from caveolin 1 and cavin proteins in each complex. Other proteins identified by mass spectrometry are not shown.

We analysed the distribution of the three cavin-GFP constructs in gradients from cells that had been cross-linked with DSP prior to cell lysis. Upon cross-linking, all cavin-GFP fusion proteins, endogenous cavin 1 and cavin 3, as well as caveolin 1 co-fractionated in the 80S fractions 8–10 (Figure 2A and 2B). Cavin-2-GFP exhibited an additional minor peak in fraction 3, and its expression led to the dissociation of small amounts of cavin 1 from the caveolar coat complex into fractions 5–7. In contrast, cavin-1-GFP and cavin-3-GFP were exclusively found in the caveolar coat complex. These data show that the cavin-GFP fusion proteins are incorporated into the caveolar coat complex just like endogenous cavins. To check that the coat complex does not reflect association or cross-linking of the cavin proteins after lysis, HeLa cell lines stably transfected with either cavin-3-GFP or cavin-3-mCherry were grown in the same dish, cross-linked, and lysed. Subsequent immunoisolation with anti-GFP antibodies yielded complexes devoid of cavin 3-mCherry (Figure S3C), arguing that cavin complexes do not form after cell lysis.

We asked whether the composition and stoichiometry of the complex is the same whichever cavin is used to isolate it. Western blotting of the complex, immuno-isolated from pooled fractions 8–10 (HMW fractions) of the gradients separately, from each cavin-GFP cell line showed that the isolated complexes are indeed indistinguishable with respect to the relative amounts of cavin 1 and caveolin 1 present in each complex (Figure 2C). To further demonstrate that the additional caveolar proteins pacsin 2 and EHD2 [27]–[30] are excluded from the coat complex, we carried out Western blotting of the isolated complex from each cavin-GFP cell line. As predicted by the mass spectrometry data (File S1), pacsin 2 and EHD2 could not be detected in isolated caveolar coat complexes (Figure 2C).

Tandem mass spectrometry was used to further characterise the caveolar coat complex isolated from each cavin-GFP and the caveolin-1-GFP cell line. All of the core caveolin and cavin proteins were identified, and in line with our Western blotting data, peptides corresponding to cavin 1, cavin 3, and caveolin 1 were found in approximately equal numbers in all four immuno-isolates (Figure 2D). Cavin 1 peptides were slightly more abundant in the cavin-1-GFP cell line, and cavin 2 peptides were notably more abundant in the cavin-2-GFP cell line—consistent with overexpression of this latter fusion protein. These mass spectrometry data, coupled with the Western blot analysis in Figure 2A and 2C, show that the composition of the caveolar coat complex is constant whichever component is used for immuno-isolation, which in turn implies that the caveolar coat complex represents one specific species of macromolecular assembly.

### The Caveolar Coat Complex Contains Cavins and Caveolins at Defined Stoichiometry, but Cavin 2 and Cavin 3 Compete for Binding Sites

**Figure 3:**
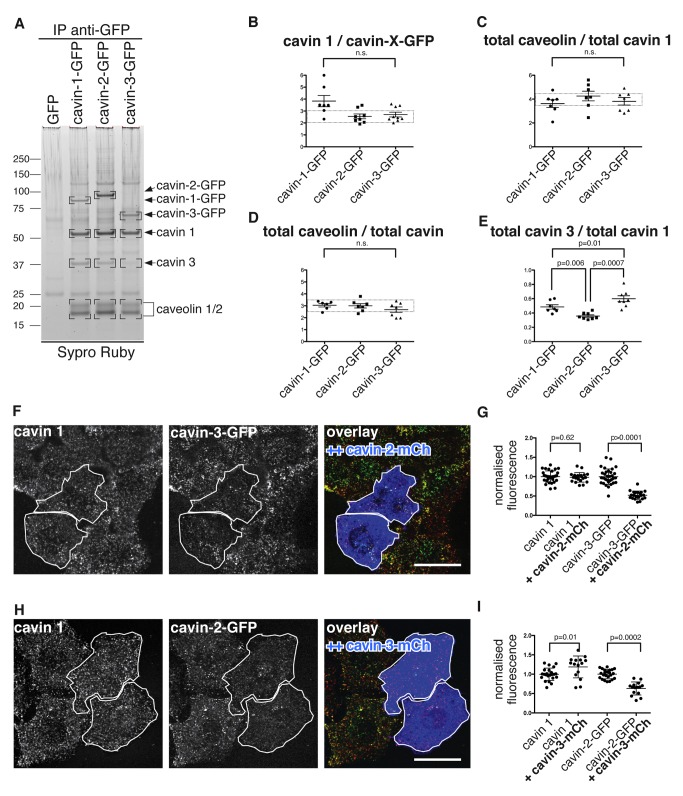
The caveolar coat complex has a defined stoichiometry. (A) Cells expressing GFP, cavin-1-GFP, cavin-2-GFP, or cavin-3-GFP were cross-linked and lysed in 1% Triton X-100/1% octyl glucoside, followed by immuno-isolation of the complexes using anti-GFP antibodies. Eluted proteins were separated by SDS-PAGE and stained with Sypro Ruby. (B–E) Quantification of the relative molar ratios between cavin 1, 2, -3-GFP, cavin 1, cavin 3, and caveolins in the caveolar coat complex. Shown are the means +/− standard error determined from 6–8 independent experiments. Student *t* test was used to determine significant differences between samples. (F) Confocal microscopy to show levels of cavin 1, detected by indirect immunofluorescence, and cavin-3-GFP, in cells overexpressing high levels of cavin-2-mCherry. Cavin-3-GFP is stably transfected in these cells. Cavin-2-mCherry is shown in blue in the overlay panel, and the same cells are outlined in the other panels. (G) Quantification of fluorescence intensity from cells as in (F). Cavin 1 and cavin-3-GFP intensities are normalised so that the mean signal in cells without cavin-2-mCherry is always 1. Data from a single experiment are shown, each data point representing a single cell. The experiment was repeated three times with equivalent results. (H and I) These represent essentially the same experiments as in (F) and (G), except that here the effect of overexpressing cavin-3-mCherry in cells stably transfected with cavin-2-GFP was examined.

We sought to determine the stoichiometry of the components of the caveolar coat complex. To this end, the complex was directly isolated from lysates of cross-linked cells, again using immuno-precipitation of either cavin-1-GFP, cavin-1-GFP, or cavin-3-GFP from the relevant cell lines. Isolated complexes were separated by SDS-PAGE, and stained with the quantitative protein dye Sypro Ruby. Cavin-1-GFP, cavin-2-GFP, cavin-3-GFP, cavin 1, cavin 3, and caveolin 1 (and caveolin 2, which is not well resolved from caveolin 1) were clearly visible in such gels, with little background from contaminating proteins (Figure 3A). We used densitometric gel scans of each immuno-precipitate from at least six separate gels and experiments to measure the relative amount of each component present. Molecular weights calculated from amino acid sequence were used to derive an estimate of the relative molar ratios between exogenously expressed cavin-GFPs, endogenous cavin 1 and cavin 3, and endogenous caveolin for all immuno-isolates (Figure 3B–E). Firstly, we found that both cavin-2-GFP and cavin-3-GFP are present in the complex at a molar ratio of slightly less than 1∶3 with cavin 1 (Figure 3B), so cavin 1 is clearly the most abundant of the three cavins. Secondly, we calculated a molar ratio of total cavin 1 to total caveolin of 1∶4 (Figure 3C)—that is, one cavin 1 may bind to four caveolin molecules (the analysis does not discriminate between caveolin 1 and caveolin 2). This ratio was constant whichever cavin was used for immuno-isolation of the complex. Thirdly, the ratio of the total amount of cavin (i.e., cavin 1+cavin 2+cavin 3) to caveolin was also constant whichever cavin was used for immuno-isolation (Figure 3D). This suggests that there are a fixed number of binding sites in the complex.

If the ratio between total cavin and caveolin in the caveolar coat complex is fixed, then one would predict that overexpression of one cavin may reduce the abundance of another cavin within the complex. Indeed, the amount of cavin 3 present when the complex was immuno-isolated from cells overexpressing cavin-2-GFP was significantly reduced compared to that observed when cavin-1-GFP or cavin-3-GFP was used for immuno-isolation (Figure 3E). This implies that cavin 2 and cavin 3 may compete for binding sites within the coat complex. In order to test this, we examined the effects of overexpressing cavin-2-mCherry and cavin-3-mCherry at high levels, using immunofluorescence to assay whether overexpression perturbs the distribution of other cavins. Overexpressing cavin-2-mCherry caused a loss of cavin 3 from caveolar puncta without perturbing the distribution of cavin 1 (Figure 3F and 3G), and overexpressing cavin-3-mCherry caused loss of cavin 2, again without altering the distribution of cavin 1 (Figure 3H and 3I). Therefore, cavin 2 can displace cavin 3 from the caveolar coat complex, and *vice versa*. Variation in the relative amounts of cavin 2 and cavin 3 occurs between tissues *in vivo* [22],[34], so this competition is likely to have physiological relevance.

The combined data imply that the single species of caveolar coat complex is composed of a defined number of cavins and caveolins. The core interaction between cavin 1 and caveolin occurs with a stoichiometry of around 1 cavin 1∶4 caveolin molecules. Changes in relative abundance imply that cavin 2 and cavin 3 compete for a defined number of binding sites within this single type of large 80S complex.

### Cavins 2 and 3 Form Distinct Subcomplexes with Cavin 1 and Caveolin 1

**Figure 4:**
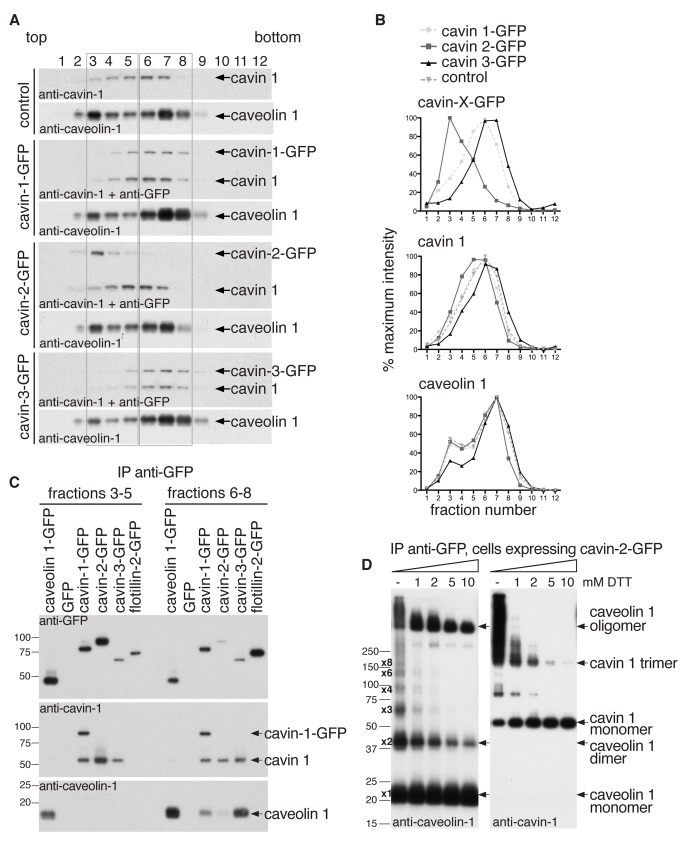
Cavin 2 and cavin 3 form distinct subcomplexes with cavin 1 and caveolin 1. (A) Gradient fractions (10–40% sucrose) of non-cross-linked and detergent solubilised (1% Triton X-100) HeLa cell extracts were analysed by Western blotting. Gradients were prepared from cells expressing either GFP (as control cells), cavin 1-GFP, cavin 2-GFP, or cavin 3-GFP. Membranes were probed with antibodies against caveolin 1, cavin 1, or GFP. Pooled fractions 3–5 and 6–8 used for immuno-isolation shown in (C) are boxed. (B) Quantification of the distribution of cavin 1, 2, and-3-GFP, cavin 1, and caveolin-1 in velocity gradients as shown in (A). Relative protein amounts were determined by densitometry of Western blots. The data are expressed as an average percentage of protein in each fraction calculated from four independent experiments. (C) Cavin 1, 2, and -3-GFP, flotillin-2-GFP, caveolin-1-GFP, or GFP were immuno-isolated from gradient fractions 3–5 or 6–8 as shown in (A). Eluted proteins were analysed by Western blotting using antibodies against GFP, cavin 1, or caveolin 1. (D) Caveolar coat complexes immuno-isolated from cross-linked HeLa cells stably expressing cavin-2-GFP were incubated with increasing concentrations of DTT to partially reduce crosslinks and analyzed by Western blotting using anti-caveolin 1 or anti-cavin 1 antibodies. Monomeric and oligomeric species of caveolin 1 and cavin 1 are indicated. Note the 180 kDa cavin 1 trimer.

Given the above, we reasoned that the 80S caveolar coat complex might be constructed from specific cavin and caveolin subcomplexes. Partially disassembled coat complexes could yield additional information on the nature of such subcomplexes. To pursue this possibility, we quantified the distribution of each cavin fusion protein in sucrose velocity gradients from the appropriate cell lines after lysis with 1% Triton X-100 *without* prior cross-linking. We observed a bimodal distribution for caveolin 1 in all cell lines, with a minor peak in fraction 3 and a major peak in fraction 7 (Figure 4A and 4B). We suggest that this latter caveolin 1 peak is the 70S species identified previously [35]. Cavin-1-GFP co-fractionated with endogenous cavin 1, as expected, and formed complexes of about 60S [35]. Cavin-1-GFP expression had no effect on the distribution of endogenous cavin 1 and caveolin 1, as compared to control cells expressing GFP alone. Interestingly, however, cavin-2-GFP peaked in fraction 3, whilst cavin-3-GFP peaked in fractions 6 and 7. Moreover, expression of cavin-2-GFP resulted in a shift of endogenous cavin 1 towards low molecular weight fractions, while cavin-3-GFP expression caused cavin 1 to shift towards high molecular weight fractions. This implies that cavin 2 and cavin 3 form distinct subcomplexes with cavin 1, with the former being smaller or less stable in detergent than the latter. Affinity purification of cavin-GFP complexes from gradient fractions 3–5 or 6–8 confirmed this idea (Figure 4C). Cavin-2-GFP was much more abundant in the low molecular weight pool 3–5, while the amounts of cavin-1-GFP and cavin-3-GFP isolated from the two pools were approximately equal. In addition, while all cavin-GFP molecules co-immunoprecipitated endogenous cavin 1, interactions with caveolin 1 were only observed in fractions 6–8, and much more caveolin 1 associated with cavin-1-GFP and cavin-3-GFP than with cavin-2-GFP. We conclude that cavin 2 and cavin 3 form separate subcomplexes with cavin 1 that are distinct in terms of size and/or stability, as well as their affinity for caveolin 1.

If cavin 2 and 3 do indeed form separate subcomplexes with cavin 1, then one would predict that cavin 1 may form complexes with cavin 2 or 3 even in the absence of caveolin 1, and that cavin 2 and 3 should not co-precipitate unless cavin 1 is present. We carried out experiments to test these predictions. There is a marked reduction in the expression of cavin 2 and cavin 3 in *cavin 1* or *caveolin 1* gene knockout mice and cell lines [23],[24],[31],[34], so we transiently transfected plasmids for overexpressing cavins 1, 2, or 3 as mCherry or GFP fusion proteins into mouse embryonic fibroblasts (MEFs) from *cavin 1* and *caveolin 1* knockout mice [7],[31],[34]. Western blotting of cell lysates from the transfected cells showed that the fusion proteins of all three cavins could be detected, although expression of cavin-3-mCherry was very low unless cavin-1-GFP was also present (Figure S5A). In *caveolin 1* knockout MEFs, immuno-isolation of cavin-1-GFP co-precipitated cavin-2-mCherry and cavin-3-mCherry (Figure S5A). This is consistent with previous studies showing that cavins form high molecular weight complexes in the absence of caveolin 1, and that cavins 1 and 2 bind to each other directly *in vitro* [21],[22],[34]. In order to ascertain whether cavins 2 and 3 can interact without cavin 1, we compared co-precipiation of cavin-2-GFP and cavin-3-mCherry in control and *cavin 1* knockout MEFs (Figure S5B). In control MEFs co-precipitation was detected, but this was lost when cavin 1 was absent, arguing that cavin 2 and cavin 3 do not interact directly, and so do indeed form separate subcomplexes with cavin 1.

To further identify protein–protein interactions within the caveolar coat complex, we used Western blotting to look for interactions between the isolated components after immunoisolation of cavin-1-GFP, cavin-2-GFP, or cavin-3-GFP followed by titration of DTT to partially dissociate cross-links. Complexes purified by isolation of cavin-2-GFP are shown in Figure 4D, and immunoisolation of all three cavins is shown in Figure S6. We found that the disassembly of cross-linked complexes with titration of DTT is precisely the same whichever cavin is used for immuno-isolation, providing additional confirmation that there is one single type of caveolar coat complex. Under nonreducing conditions (without DTT), around 50% of the total caveolin 1 in the caveolar coat complex was found in oligomers of about 350 kDa and more. The remainder of the caveolin 1 was present mostly as either monomers or dimers (Figure 4D). Upon titration of DTT, high molecular weight oligomeric forms of caveolin 1 were reduced to a distinct caveolin oligomer of about 350–400 kDa, which was stable in up to 10 mM DTT and even clearly identifiable under fully reducing conditions (not shown). More minor cross-linked species consistent with the presence of caveolin 1 oligomers increasing in size from 2 to 8 caveolin 1 monomers were not stable in DTT. This suggests that the 350–400 kDa oligomeric form of caveolin 1 is a major component of the caveolar coat complex [49],[50]. We then analyzed cavin 1 (Figure 4D and Figure S6). In nonreduced samples, the large majority of cavin 1 was cross-linked into oligomers of about 180 kDa and more. Monomeric cavin 1 (55 kDa) and a minor cavin 1 species of about 85 kDa were also observed. Interestingly, progressive addition of DTT revealed a relatively stable oligomeric form of cavin 1 of about 180 kDa, a size indicative of a cavin 1 trimer. This form of cavin 1 was found in all immunoisolates and was stable in up to 10 mM DTT (Figure 4D and Figure S6).

Altogether, combining the data on disassembly of the non-cross-linked caveolar coat complex in detergent, co-precipitation in the absence of cavin 1, and partial reduction of cross-links yields specific conclusions: Subcomplexes containing cavin 1 and cavin 2 can be separated from subcomplexes containing cavin 1 and cavin 3, and cavins 2 and 3 do not enter the same complex unless cavin 1 is also present. Partial reduction of cross-links shows that cavin 1 forms a relatively stable trimer, and this trimer is likely to be a core element of the caveolar coat complex.

### The Ratio Between Cavin 1 and Caveolin 1 in the Coat Complex Is Independent of Cavin 2, Cavin 3, and EHD2 Expression

**Figure 5:**
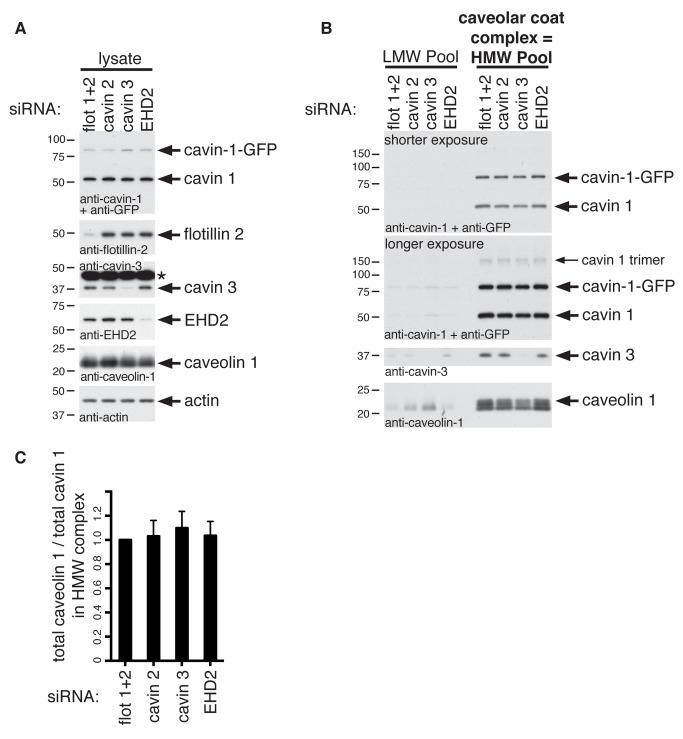
A core complex containing cavin 1 and caveolin 1 is independent of cavin 2, cavin 3, and EHD2 expression. (A) Western blotting of cell lysates from siRNA-treated cells. Blots were probed with the anitbodies indicated. * indicates the nonspecific band obtained with anti-cavin-3 antibodies. (B) Western blotting of fractions 3 to 5 (LMW pool) and 8 to 10 (HMW pool: these fractions contain the caveolar coat complex) of sucrose velocity gradients analyzing the distribution of cavins and caveolin 1 from cells treated with the siRNAs shown. Blots of the entire gradients are shown in Figure S7B. (C) Quantification of the ratio between the amount of cavin 1 and caveolin 1 in fractions and 8 to 10 (HMW pool: these fractions contain the caveolar coat complex) of sucrose velocity gradients as shown in the blots in (B). Quantification is from densitometric scans of Western blots, and is based on three separate experiments; bars are SEM. In each experiment, all values were normalized so that the intensity of relevant bands in control flotillin 1 and 2 siRNA treated samples was 1.

So as to characterise the role of cavins 2 and 3 in the caveolar coat complex in more detail, we used siRNAs to deplete these proteins from the cavin-1-GFP cell line. In parallel, we also used siRNAs targetting EHD2, to ask whether this protein controls the assembly of the complex, and siRNAs targetting flotillin proteins as a negative control [51]. Depletion of all targeted proteins was highly efficient, as judged by Western blotting (Figure 5A and Figure S7A). Velocity gradient centrifugation showed that 80S complexes were clearly still present in all siRNA-treated cells, and the large majority of cavin 1, cavin 3, and caveolin 1 were still found in the HMW, 80S, fractions 8–10 (Figure 5B and Figure S7B). Lack of cavin 3 caused a marginal destabilisation of the 80S complex, as in this case some cavin 1 and caveolin 1 were detected in lower density fractions in longer exposures of the relevant Western blots (LMW pool, Figure 5B and Figure S7B). In these longer exposures the cavin 1 trimer described above is detected, even though these samples were run under fully reducing conditions. Most importantly, however, quantification of the ratio between the amounts of total cavin 1 and caveolin 1 present in the high molecular weight fractions (i.e., in the caveolar coat complex) confirmed that this was unchanged by any of the siRNA treatments (Figure 5C), which shows that the stoichiometry of a core interaction between cavin 1 and caveolin 1 is independent of the presence of cavins 2 or 3.

Likewise, depletion of EHD2 had no detectable effect on the formation of the caveolar coat complex, having no effect on the behaviour of the complex in velocity gradients or on the relative amounts of cavin and caveolin proteins present (Figure 5B and 5C, Figure S7). EHD2, therefore, not only is not present in the complex, but also does not regulate its formation. Given that EHD2 controls the dynamics and plasma membrane association of caveolae [27],[28], this implies that the caveolar coat complex is the same whether caveolae are continuous with the plasma membrane or form intracellular membrane vesicles.

### Cavins 1, 2, and 3, and Caveolin 1 Are Uniformly Distributed Around the Caveolar Bulb

**Figure 6:**
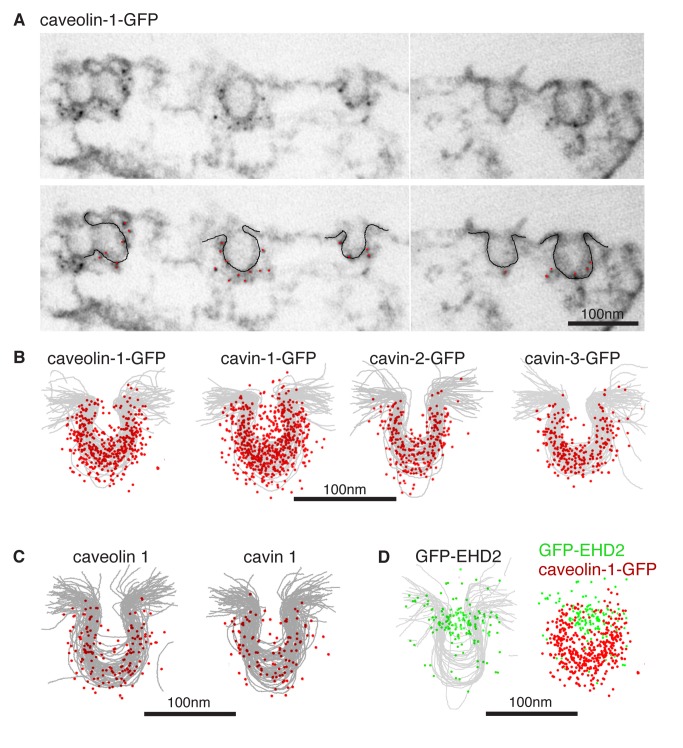
Cavins and caveolins are uniformly distributed around the caveolar bulb. (A) HeLa cell lines stably transfected with either caveolin-1-GFP, cavin 1, 2, or-3-GFP were processed for immuno-electron microscopy, using anti-GFP primary antibodies, nanogold-conjugated secondary antibodies, and silver enhancement. Representative transmission EM images showing specific immuno-labeling of caveolin-1-GFP. (B) Membrane profiles and the localisation of gold particles (as shown in A) from about 50 caveolae per cell line as shown were superimposed; labeling was with anti-GFP antibodies as in (A). (C) HeLa cells were processed for immuno-electron microscopy using anti-caveolin 1 or cavin 1 primary antibodies, nanogold-conjugated secondary antibodies, and silver enhancement. Images represent superimposition of around 50 caveolae. (D) Membrane profiles and the localisation of gold particles (as shown in A) from about 50 caveolae from GFP-EHD2 expressing cells were superimposed; labeling was with anti-GFP antibodies as in (A).

If the caveolar coat complex does indeed coat caveolae, then it should be found all around the caveolar bulb. We aimed to determine the localisation of the complex within caveolae at high spatial resolution. Firstly, the cell lines expressing caveolin-1-GFP, cavin-1-GFP, cavin-1-GFP, and cavin-3-GFP described above were studied by immuno-electron microscopy. Pre-embedding labeling using anti-GFP antibodies and nanogold-conjugated secondary antibodies, followed by silver enhancement, allowed highly specific labeling of caveolae (Figure 6A). We acquired images of more than 50 caveolae stained with gold particles per cell line, and superimposed both the membrane profiles and the position of gold particles for each case (Figure 6B and Figure S8). The aggregated images revealed that all of the cavin-GFP fusions, as well as caveolin-1-GFP, localised around the caveolar bulb, with no discernable bias towards the membrane proximal or distal region. In order to check that endogenous proteins have the same distribution as GFP fusions, we carried out immuno-labeling of untransfected cells with caveolin 1 and cavin 1 antibodies. The distribution of endogenous caveolin 1 and cavin 1 determined using this approach was indistinguishable from that of caveolin-1-GFP and cavin-1-GFP (Figure 6C). These data argue that caveolar coat complexes are distributed all around the membrane bulb of caveolae.

Our biochemical data show that EHD2 is not present in the caveolar coat complex. To determine the distribution of EHD2 within caveolae, we used cells expressing GFP-EHD2 and immuno-labeling as above (Figure 6D). In clear contrast to the caveolar coat complexes, GFP-EHD2 was enriched around the neck of caveolae (Figure 6C). Therefore, caveolae are likely to have separate sets of proteins coating the bulb and neck regions, and these different distributions can be resolved by immuno-electron microscopy.

Immuno-labeling has inherent limitations, including the possibility that epitopes may not be uniformly accessible or may only tolerate weak fixation, and the fact that complexes of primary and secondary antibodies reduce spatial resolution compared to the fine structural details otherwise delivered by electron microscopy. We aimed to directly visualise caveolar coat complexes by electron microscopy *in situ*. MiniSOG (for Mini Singlet Oxygen Generator) is a relatively small (106 amino acids) fluorescent flavoprotein that efficiently generates reactive oxygen species upon illumination with blue light. Local production of reactive oxygen species can be used to convert diaminobenzidine (DAB) into an osmiophilic electron-dense polymer. This allows proteins to be localised by EM at high spatial resolution [48],[52]. We generated separate cell lines expressing cavins 1, 2, and 3 as MiniSOG-mCherry fusion proteins (henceforth referred to as cavin-MiniSOG). In order to facilitate analysis of multiple caveolae, we used retinal pigment epithelial (RPE) cells, where caveolae polarise to the rear of the cell, as seen in other polarized cells such as fibroblasts (Figure S9B) [41],[53]. This yields defined regions of the cell that are very rich in caveolae. Cavin fusion proteins were expressed at low levels relative to the endogenous proteins, and localised to caveolae in a manner indistinguishable from the endogenous proteins by light microscopy (Figure S9A, Table S1).

**Figure 7:**
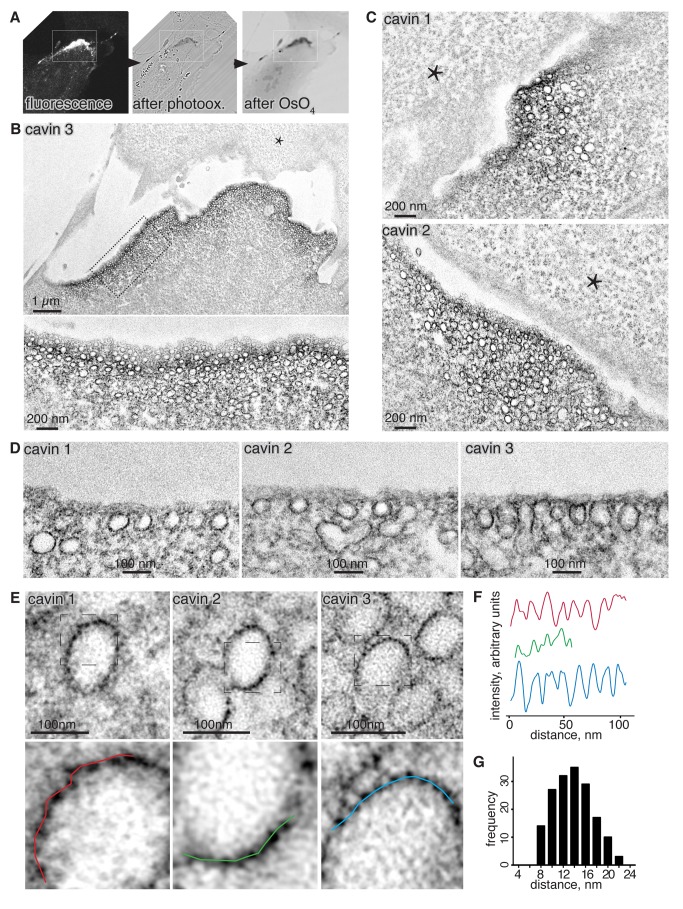
MiniSOG labeling shows that cavins are distributed around the caveolar bulb, with periodic density maxima. RPE cells stably transfected with cavin 1, 2, or 3-MiniSOG-mCherry were photooxidized and processed for correlative light and electron microscopy. (A) Representative light microscopy images showing an RPE cell expressing cavin-3-MiniSOG-mCherry before (left) and after photooxidation (middle), and after osmification and plastic embedding (right). (B) Correlative electron micrograph of the photooxidized region shown in (A). The bottom electron micrograph shows the boxed region shown in (B), acquired at higher magnification. (C) Representative transmission electron micrographs of RPE cells stably expressing cavin-1-MiniSOG-mCherry (top) and cavin-2-MiniSOG-mCherry (bottom). (D) Representative electron micrographs for all three cavin-MiniSOG-mCherry showing caveolae visibly open to the outside of the cell. Note that MiniSOG label is found all around the caveolar bulb. Asterisks highlight background contrast produced by osmium stain in adjacent cells. (E) Representative electron micrographs of photooxidized regions of RPE cells stably transfected with cavin 1, 2, or 3-MiniSOG-mCherry. Areas showing regular spacing between densities produced by MiniSOG are boxed and shown enlarged on the bottom. (F) Line scans illustrating periodic density maxima as shown in (E). (G) Quantification of the distances between density maxima produced by cavin-MiniSOG-mCherry (*n* = 176).

Photooxidation of glutaraldehyde-fixed cavin-MiniSOG expressing RPE cells in the presence of DAB resulted in the deposition of a brown reaction product (Figure 7A). Correlative electron micrographs of plastic embedded and osmium-stained sections revealed a high density of caveolae in such regions (Figure 7B). Caveolae were strongly labeled with an electron-dense stain, regardless of which cavin-MiniSOG was used for photooxidisation (Figure 7B and 7C). The staining was highly specific, as caveolar membranes from adjacent cells not expressing cavin-MiniSOG were not stained, the stain was restricted to regions of the cell enriched in caveolae, and the only cellular membranes stained were caveolae (Figure 7 and Figure S10). MiniSOG-generated stain from all three cavin fusion proteins was distributed right around the caveolar bulb, and was present at the same density at the lateral sides and at the apex of the bulb (Figure 7D). This is consistent with the immuno-electron microscopy presented in Figure 6, and again implies that caveolar coat complexes are present all around the caveolar bulb.

### Ultrastructure of the Caveolar Coat

We acquired high-resolution micrographs to study the ultrastructural properties of the caveolar coat complex labeled using cavin-MiniSOG. In thin sections, the label was clearly not continuously distributed along the caveolar membrane, but rather formed a punctate, sometimes spike-like coat. This was observed for all cavin-MiniSOG proteins (Figure 7E). In some caveolae, individual puncta formed periodic densities with approximately regular spacing (Figure 7E and 7F). The spacing of periodic densities around the bulb was not measurably different whether cavin-1-MiniSOG, cavin-2-MiniSOG, or cavin-3-MiniSOG were used (Figure 7F). Quantification of the spacing of these local increases in density revealed a periodicity of 10–16 nm. The shortest distance we were able to measure in electron micrographs of thin sections was around 8 nm (Figure 7G). This shows that caveolar coat complexes form local densities on caveolar membranes with an apparent spacing of 10–16 nm and that MiniSOG labeling allows proteins to be localized with low nanometer precision. The fact that we could observe regular spacing between densities is suggestive of regularity in the coat.

**Figure 8:**
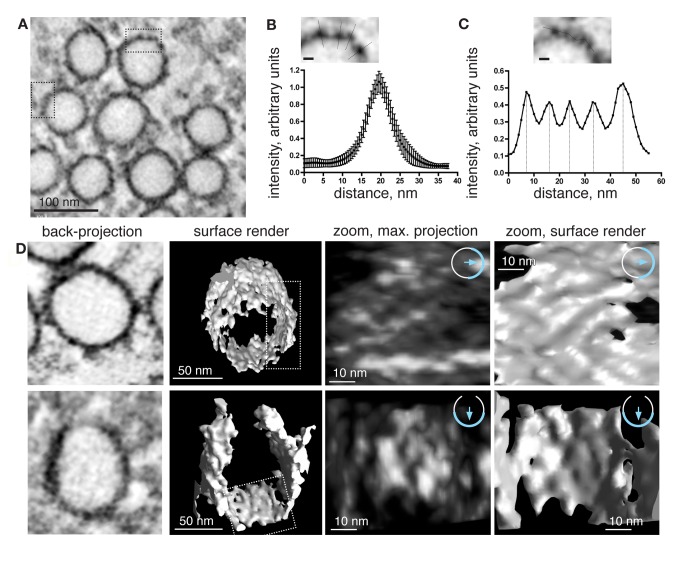
Tomographic reconstruction of the caveolar coat stained with cavin-3-MiniSOG. Tomograms were recorded from representative regions of photooxidized RPE cells stably transfected with cavin-3-miniSOG-mCherry. (A) x/y projection of a typical tomogram acquired at 150 kV, 18k at a pixel size of 0.5 nm. The backprojection shown was binned by 2, resulting in a pixel size of 1 nm. (B and C) Line scans through density maxima observed in x/y projections. (B) Line scans were performed perpendicular to the membrane and the relative intensity plotted against distance, including error bars (*n* = 11). (C) Representative line scan along the membrane showing regular spacing between local maxima. Note that distances of about 8–10 nm can be resolved. (D) Three-dimensional representation of the caveolar coat stained with cavin-3-MiniSOG, for two separate caveolae. From left to right: x/y backprojections of representative caveolae; surfaces of the caveolae shown on the left; maximum intensity projection of the boxed area shown in column 2; surface rendering of the boxed area shown in column 2.

In order to reveal the organisation of the coat in three dimensions, dual-tilt tomograms were recorded from representative regions. Tomography confirmed that cavin-MiniSOG labeling extends all around the bulb (Figure 8A and Movie S1), and that caveolar coat complexes form periodic density maxima (Figure 8B and 8C). In order to estimate the degree of resolution of the MiniSOG label in our tomograms, line scans through individual densities were performed. Line scans perpendicular to the membrane showed that densities peaked sharply and exhibited a half maximum width of about 8–10 nm (Figure 8B). Line scans along caveolar membranes resolved densities separated by about 10 nm, confirming our previous data on thin sections (Figure 8C: compare to 7F). We carried out three-dimensional reconstructions of regions of the caveolar surface where such periodic density maxima were well resolved (Figure 8D). These reconstructions revealed both local maxima and apparent linear striations within the coat (Figure 8D and Movie S2). In some regions, the density maxima had a regular, lattice-like distribution, suggesting that the distribution of MiniSOG reflects an underlying higher-order lattice organisation of the caveolar coat. The three-dimensional reconstructions also reinforced the firm conclusion that the MiniSOG label, and hence the caveolar coat complex, are found all around the caveolar bulb without specific enrichment in the sides, apex, or neck of caveolae.

## Discussion

We show that caveolins and cavins can be purified as a single species of protein complex that excludes EHD2 and pacsin 2. We term this complex the caveolar coat complex. Purification of the caveolar coat complex, and a comprehensive quantitative analysis of its composition, allows us to put forward a model of the basic stoichiometry. Cavin 1 is a core component of the complex, and several independent experiments provide evidence for cavin 1 forming a trimer: Firstly, we identified a 180 kDa cavin 1 species, a size compatible with a trimer, which was relatively stable. Secondly, cavin 2 and cavin 3 interact with cavin 1 at a molar ratio of about 1∶3, suggesting that one cavin 2 or cavin 3 molecule associates with the cavin 1 trimer. Thirdly, cavin 1 is predicted to form a three-stranded coiled coil via its N-terminal domain (http://groups.csail.mit.edu/cb/multicoil/cgi-bin/multicoil.cgi) [54]. We therefore suggest that the core of the coat complex is composed of a cavin 1 trimer. For each cavin 1 molecule in the complex, there are likely to be 4 caveolins. Both in our experiments and previously [49],[50],[55], an SDS-resistant oligomeric state of caveolin 1 has been detected, with an apparent molecular mass of 350–400 KDa. Although this seems slightly too large to be the 12 caveolins predicted by our stoichiometric measurements and the presence of cavin 1 trimers, it is possible that SDS-resistant complexes run anomalously on SDS-PAGE due to pronounced secondary or tertiary structure. Our data imply that the basic unit of 1× cavin 2 or 3∶3× cavin 1∶12× caveolin must assemble into larger multimers to generate the 80S caveolar coat complex. Whether the 80S complex represents all of the coat present on an individual caveolar bulb or an intermediate level of structural organisation is not yet clear.

Previous studies have shown that cavins and caveolin 1 in detergent-solubilised lysates fractionate differently on gradients and do not efficiently co-precipitate, which could be interpreted as arguing for lack of interaction in intact cells [22],[35]. Although our cross-linking data strongly suggest that cavin 1 and caveolin 1 do in fact interact, it will be important to demonstrate this directly. Higher resolution structural information on cavins and caveolins individually and in complexes is required. This may allow elucidation of the precise nature of the molecular contacts made between cavin 1 and caveolin 1. Nevertheless, it is clear that a core complex containing cavin 1 and caveolin 1 is not dependent on the presence of cavins 2 or 3, as the ratio between cavin 1 and caveolin 1 in this complex does not change when cavin 2 or cavin 3 are not present.

Biochemical and imaging experiments argue that cavin 2 and cavin 3 compete for binding to the cavin 1 trimer, as cavin 2 can displace cavin 3 from the complex and *vice versa*. This implies that, when cavin 2 and cavin 3 are both present in the same cell, the 80S caveolar coat complex will contain both cavin 1 trimers bound to cavin 2, and cavin 1 trimers bound to cavin 3. The observations that cavin 1 will co-precipitate cavin 2 or cavin 3 even in the absence of caveolin 1, but cavins 2 and 3 do not co-precipitate in the absence of cavin 1, are consistent with this. We hypothesise that it is the balance between cavin 2 and cavin 3 that confers additional structural and functional properties on the coat complex. Either overexpression of cavin 2 or siRNA-mediated knockdown of cavin 3 caused a slight but measurable dissociation of cavin 1 and caveolin 1 from the coat complex. Moreover, cavin 2 and cavin 3 formed separate complexes with cavin 1 under noncrosslinking conditions, with the former being smaller or less stable than the latter. These combined data suggest that shifting the ratio between cavin 2 and cavin 3 within the 80S complex towards having relatively more cavin 2 could make the complex less stable, and *vice versa*. This might be a means to modulate caveolar functions in different tissues, as the ratio between cavin 2 and cavin 3 varies *in vivo* [22],[34], and the presence of cavin 2 is associated with apparently smaller cavin complexes *in vivo* [34]. Therefore, the findings presented here correlate with, and imply molecular explanations for, the *in vivo* data. Further *in vivo* experiments will be needed to address the functional and physiological consequences of the variation of the cavin 2 and cavin 3 complement within the caveolar coat complex.

Both MiniSOG-tagged cavins and immuno-EM show that the caveolar coat complex is found all around the caveolar bulb, while the caveolar neck is likely to contain additional proteins including EHD2. Notably, our data do not provide any evidence for EHD2 making direct contact with the caveolar coat complex [27],[28], leading to the concept that the neck region constitutes a separate subdomain within caveolae that is distinct from the rest of the bulb [41]. The observation that siRNAs that efficiently target expression of EHD2 have no effect on the size or composition of the caveolar coat complex provides additional evidence that EHD2 has a separate role within caveolae. The previous finding that EHD2 controls the plasma membrane association and dynamics of caveolae [27],[28] leads to the additional conclusion that the coat complex is likely to be the same whether caveolae exist as characteristic flask-shaped invaginations of the plasma membrane, or as intracellular membrane vesicles.

The production of reactive oxygen species by MiniSOG, and consequent deposition of osmiophilic DAB polymer, provides a highly specific label for electron microscopy [48], and our data highlight its utility as a probe for cell biological structures. Local maxima in density produced by MiniSOG are likely to reflect increased local concentration of the tag and hence the tagged protein. Our images suggest that diffusion of reactive oxygen and DAB product away from the MiniSOG is limited, as line scans across the consequent electron density reveal that periodic changes in density over a distance scale of 10 nm can be clearly resolved. Nevertheless, diffusion of singlet oxygen or DAB reaction product is likely to provide a limit to the resolution of MiniSOG generated label. The ultimate resolution achievable by the MiniSOG molecular contrasting system remains to be determined, but structural details measuring a few nanometers have been observed [48].

We show that the caveolar coat complex generates a lattice on the surface of caveolae that can be detected using MiniSOG fusions, as local density maxima in thin sections and in tomographic reconstructions of the caveolar surface. We present evidence from both TEM on thin sections and 3D tomography that the coat is regular, with a periodic spacing of 10–15 nm. Ridges or striations can be observed, as shown in Figure 8D, and some regions of the surface show regular arrays of maxima. Our data, however, do not completely resolve the high-resolution internal geometry of this lattice. It is possible that regions containing less well defined densities and occasional gaps in the lattice are due to local cellular factors restricting or enhancing diffusion of the MiniSOG-produced electron dense stain. It is also possible that the caveolar coat is not as well ordered or arrayed as, for example, clathrin-coated pits. Nevertheless, the key point is that the coat represents a single type of complex coating the caveolar bulb, rather than previously plausible alternative possibilities such as cavins and caveolins being present in different complexes with different distributions.

The observation of local maxima in MiniSOG density with a spacing of around 10–15 nm agrees very well with local density maxima around the caveolar bulb in ultrathin plastic sections prepared from samples stained conventionally [41], and with the spacing of striations on the surface of caveolae revealed by platinum coating of membrane fragments [6],[43]. It is therefore likely that the caveolar coat complex described here is responsible for previously reported, but molecularly undefined, ultrastructural features on the surface of caveolae. Our data, revealing the identity, basic stoichiometry, and distribution of this unitary complex around the caveolar bulb, open the way for further structural characterisation of the protein machinery for generating caveolae.

## Materials and Methods

### Antibodies

The following antibodies were used: Mouse anti-GFP (Roche, 11814460001), rabbit anti-PTRF (cavin 1) (Abcam, ab48824), rabbit anti-SRBC (PRKCDBP; cavin 3) (Abcam, ab83913), goat anti-SDPR (cavin 2) (R&D Systems, AF5759), goat anti-EHD2 (Abcam, ab23935), rabbit anti-Caveolin 1 (BD, 610060), mouse anti-flotillin-1 (BD, 610821), mouse anti-flotillin-2 (BD, 610384), mouse anti-clathrin heavy chain (×11), rabbit anti-GFP antibody (Abcam, ab6556), and rabbit anti-RFP (MBL, PM005).

### Protein Constructs and Cell Lines

Constructs for human PTRF-mCherry (cavin 1), human SDPR-mCherry (cavin 2), and human SRBC-mCherry (cavin 3) have been described [21]. To generate TEV-GFP-10×His fusion constructs, a TEV-GFP-10×His cassette was inserted into the BamHI/NotI sites of pClontech N1. The respective cDNAs were inserted in frame. To generate MiniSOG-mCherry cavin constructs, MiniSOG cDNA was inserted into pClontech N1 via BamHI/AgeI. Constructs were transfected into HeLa and RPE cells using FugeneHD (Promega) according to the manufacturer's recommendations. Clonal HeLa cell lines expressing cavin-TEV-GFP-10×His proteins were produced from single cell clones and cultured in DMEM, 10% FCS, penicillin/streptomycin supplemented with 0.4 mg/ml G418 (Sigma). RPE cells expressing cavin-MiniSOG-mCherry were selected by FACS and cultured in 50% DMEM/50% F12 medium, 10% FCS, 5 mM glutamine, penicillin/streptomycin, supplemented with 0.4 mg/ml G418.

### Crosslinking, Cell Lysis, and Velocity Gradient Centrifugation

For crosslinking studies, semiconfluent cultures of HeLa cells were washed twice with ice-cold PBS and incubated on ice with 1.2 mM DSP (Pierce) in ice-cold PBS for 1 h. A 100× DSP stock solution was prepared in DMSO fresh prior to use. After 1 h, DSP was quenched by addition of 1 M Tris pH 7.4 to a final concentration of 100 mM for 15 min. Cells were briefly rinsed in 100 mM Tris pH 8 and immediately scraped into lysis buffer (LB): 50 mM Tris pH 8, 300 mM NaCl, 5 mM EDTA, protease inhibitor cocktail (Roche). Dependent on the experiment, either 1% (v/v) Triton X-100, 2% (w/v) octyl-glucoside (OG), or a combination of 1% Triton X-100/1% OG were added to LB. Cell lysates were incubated on ice for 30 min and spun at 14,000 rpm in a table top centrifuge for 30 min at 4°C, followed by a second centrifugation for 10 min. Lysates were added atop a linear 10–40% (w/v) sucrose gradient prepared in LB plus 0.2% Triton X-100. Gradients were spun in a SW40Ti rotor at 37,000 rpm for 6 h at 4°C. Twelve 1 ml fractions were collected from the bottom of the gradient by tube puncture. For Western blotting, equal volumes (usually 250 µl) of each fraction were precipitated with MeOH/Chloroform. The pellet was dissolved in 1×LDS loading buffer (Invitrogen) and boiled for 2 min. Proteins were separated on NuPAGE 4–20% Tris/Glycine or 4–12% Bis/Tris gels (Invitrogen) and blotted onto PVDF membranes (Millipore).

### Immunoisolation

For immunoisolation of GFP-tagged proteins, magnetic anti-GFP microbeads, and μcolumns (Miltenyi Biotech) were used. For immunoisolation of the HMW complex from sucrose gradients, fractions 8–10 were pooled (total 3 ml) and incubated with 20 µl anti-GFP beads for 2–4 h at 4°C rotating. Alternatively, 1 ml of total cell lysate was incubated with 10 µl anti-GFP beads. Lysates were applied to μcolumns and washed eight times with 2 ml of LB/1% Triton X-100 at room temperature. A final wash was performed with LB without detergent added. Protein complexes were eluted from the column with 140 µl 0.1 M TEA, pH 11.8, immediately neutralized by addition of 70 µl 1 M Tris, pH 7.4, and subjected to tandem mass spectrometry. Alternatively, protein complexes were eluted with elution buffer (Miltenyi Biotech) and separated by SDS-PAGE.

### Stoichiometric Analysis of the Caveolar Coat Complex

Immunoisolates from total cell lysates were separated on 4–12% NuPAGE Bis/Tris gels. Gels were washed twice in distilled H_2_0 for 5 min each and stained with the fluorescent protein dye SYPRO RUBY (Lonza) for 1 h at room temperature. Gels were washed several times in distilled H_2_0 and the fluorescence scanned on a Chemidoc XRS+ Molecular Imager. The intensities of protein bands corresponding to cavin-GFP fusion proteins, cavin 1, cavin 3, and caveolins were determined using Image Lad software. Bands corresponding to alpha and beta caveolin 1 as well as caveolin 2 could not be resolved clearly and were thus quantified as one band. All values were corrected for by subtracting background fluorescence from the same molecular weight regions of GFP control samples. To calculate relative molar ratios between the protein components of the complex, the following molecular masses were used: Cavin1-GFP, 80 kDa; cavin-2-GFP, 90 kDa; cavin-3-GFP, 65 kDa; cavin 1, 55 kDa, cavin 3, 36 kDa, caveolin, 20 kDa. For documentation, data were exported to Prism Graphpad.

### siRNA Transfection of HeLa Cells

On-target Plus SMART pool siRNAs against human cavin 2, human cavin 3, and human EHD2 were from Thermo Scientific (L-015910, L-016416, and L-016660, respectively). siRNAs against human flotillin 1 and flotillin 2 (J-010636-05, J-010636-06, J-003666-09, J-003666-10) were pooled and used as a control throughout. Cavin-1-TEV-GFP-10×HIS HeLa cell lines were transfected at 30% confluency using Oligofectamine (Invitrogen) and a total of 100 nM siRNA per transfection. Four to five days posttransfection, cells were cross-linked with 1.2 mM DSP as described above and then lysed in LB/1% OG/1% Triton X-100. Cell lysates were cleared by centrifugation at 14,000 rpm for 30 min and loaded atop 10–40% sucrose gradients. Gradients were spun as described above. To quantify the relative amounts of cavin 1 and caveolin 1 in the low molecular weight (LMW; fractions 3–5) and high molecular weight fractions (HMW; fractions 8–10), equal volumes of each fraction were pooled, MeOH/chloroform precipitated, and analysed by Western blotting. The ratio of caveolin 1 to cavin 1 in the HMW pool 8–10 was calculated from three independent experiments, using densitometry in ImageJ.

### Immunoprecipitation from MEFs

Wild-type MEFs, *caveolin 1* −/− MEFs, or *cavin 1* −/− MEFs [34] were co-transfected with equal amounts of pDNA using electroporation. Different combinations of the following constructs were used: Cavin-1-TEV-GFP-10×HIS, cavin-2- TEV-GFP-10×HIS, cavin3-miniSOG-mCherry, cavin-2-miniSOG-mCherry. Per co-transfection, 1×10^6^ cells were transfected with 2.5 µg of each pDNA. Twenty-four h posttransfection, cells were cross-linked with 3 mM DSP as described above and lysed in LB/1% OG/1% Triton X-100. Lysates were cleared by centrifugation at 14,000 rpm and incubated with 10 µl anti-GFP microbeads (Miltenyi Biotec) for 2 h at 4°C. Immunprecipitates were washed five times with LB/1% Triton X-100 and eluted with 60 µl elution buffer (Miltenyi Biotec). Equal volumes were analysed by Western blotting.

### Light Microscopy

HeLa or RPE cells were fixed in 4% paraformaldehyde in PBS pH 7.4 for 10 min and stained with primary antibodies o/n in PBS, 3% FCS, 0.2% saponin. Cells were washed with PBS and incubated in secondary antibodies for 1 h. TIRF microscopy was carried out using an Olympus IX71. Confocal micrographs were captured on a ZEISS 510 LSM using standard filter sets. Co-localisation was quantifed using the Pearson correlation coefficient, as implemented in the “Colocalization Finder “plugin for Image J (http://rsbweb.nih.gov/ij/plugins/colocalization-finder.html).

### Immuno-Labeling

HeLa cells were grown on glass bottom petri dishes (MatTek) and fixed in 4% Paraformaldehyde in 0.1 M phosphate buffer pH 7.4 overnight at 4°C. After several buffer washes, followed by inactivation of reactive aldehyde groups using 0.1% sodium borohydride in phosphate buffer for 15 min, cells were permeabilised using 0.03% saponin in 20 mM phosphate buffer, 150 mM sodium chloride for 30 min. Cells were incubated in normal goat serum (Aurion) for 40 min prior to incubation in rabbit anti-GFP antibody (Abcam) used at 1∶800 for 4.5 h at room temperature. After thorough washing, cells were incubated with 1∶200 dilution of goat anti-rabbit ultrasmall gold (Aurion) overnight at 4°C. After washing cells were fixed with 2% glutaraldehyde in 0.1 M phosphate buffer for 30 min and washed with distilled water followed by silver enhancement of gold using R-Gent SE-EM (Aurion) reagents. Cells were then postfixed with 0.5% osmium tetraoxide in 0.1 M phosphate buffer on ice for 15 min. Cells were then dehydrated in an ascending ethanol series and embedded in CY212 resin. Ultrathin sections were stained with saturated aqueous uranyl acetate and Reynolds lead citrate and examined using a Philips 208 EM operated at 80 kV.

### MiniSOG Photooxidation and Electron Microscopy

For photooxidation, cells cultured in MatTec glass bottom dishes were fixed at room temperature with 2% glutaraldehyde (EM grade, EMS Corp.), 2.5 mM CaCl_2_ in 0.1 M cacodylate buffer pH 7.4 (CB), and immediately transferred onto ice for 1 h. Cells were rinsed five times with ice-cold CB and blocked with 50 mM glycine, 10 mM potassium cyanide, and 10 mM aminotriazole in CB for 15 min on ice. Cells were washed five times with CB and transferred onto a cooled stage on a Leica SPE II confocal microscope. A freshly prepared solution of 0.5 mg/ml diaminobenzidine (DAB, Sigma) in CB was added to the cells. Areas of interest were photooxidized by illumination with blue light, using a 150W xenon lamp, a standard FITC filter set, and a 63× objective NA 1.3. After about 3–4 min, a brownish precipitate formed in place of the fluorescence. Cells were removed from the stage, washed five times with CB, and poststained with 1% osmium tetraoxide in CB for 30 min at room temperature. Cells were washed five times with water, followed by dehydration in 20, 50, 70, 90, and 100% EtOH. Cells were infiltrated in Durcupan ACM resin (EMS Corp.). Photooxidized areas were sawed out of the dish and sectioned. For 2D transmission electron microscopy (TEM), 80 nm sections were sectioned. Electron micrographs were recorded at 80 or 120 kV on a FEI T12 TEM. Images were recorded with Serial EM software and a 2k×2k Gatan CCD camera.

### 3D Tomography

Three-dimensional electron tomography was carried out on 250–300 nm sections at 150 or 300 kV using a FEI Titan TEM. Sections were carbon-coated, glow-discharged, and dipped into a solution of 0.1% BSA and 5 nm colloidal gold particles. Dual tilt series were recorded at +/−60° with 1° intervals and a pixel size of 0.5 nm (at 18k). Images were captured using a 4k×4k Gatan Ultrascan 4000 camera. Reconstruction was accomplished using a combination of IMOD [56] and TxBR [57] reconstruction packages. Rough alignment of the two tilt series was done with IMOD software package, and fine alignment and reconstruction was done using the TxBR package.

## Supporting Information

Figure S1 **Cavin/caveolin complexes are sensitive to detergent, but can be stabilized by crosslinking.** HeLa cells, either cross-linked with DSP or left untreated, were solubilised in either 0.5% Triton X-100 (A), 1% Triton X-100 (B), or 2% octyl glucoside (C). Lysates were fractionated on 10–40% sucrose gradients, followed by Western blotting of gradient fractions 1–12 using antibodies against caveolin 1 or cavin 1. Without cross-linking, oligomeric complexes of caveolin 1 are sensitive to the detergent used for solubilisation. Full dissociation of caveolin oligomers was achieved with 2% octyl glucoside (C top) or a combination of 1% Triton X-100/1% octyl glucoside (Figure 1A). Note that cross-linking stabilises a high molecular weight (HMW) complex of both caveolin 1 and cavin 1 in all detergents tested.
(TIF)

Figure S2 **Isolation of the caveolar coat complex using HeLa cells stably transfected with caveolin-1-GFP.** (A) Western blots of lysates from HeLa cells stably transfected with caveolin-1-GFP, flotillin-2-GFP, or GFP. Membranes were probed with anti-GFP, anti-flotillin 2, or anti-caveolin 1 antibodies. Note that caveolin-1-GFP is expressed at low levels relative to endogenous caveolin 1, and that there is no detectable proteolysis of caveolin-1-GFP. (B) Coomassie-stained protein gel of gradient fractions 1–12 prepared from DSP-cross-linked HeLa cells. The HMW peak of fractions 8–10 that contain caveolin 1 and cavin 1 is boxed. Fractions 7–9 are rich in ribosomal proteins (dashed box). The 60S peak obtained for purified 60S ribosomal subunit is indicated. (C) HeLa cells stably transfected with caveolin-1-GFP, flotillin-2-GFP, or GFP were cross-linked with DSP and lysed in 1% Triton X-100/1% octyl glucoside. Lysates were fractionated on 10–40% sucrose gradients and pooled HMW fractions 8–10 used for immuno-isolation of the caveolar coat complex. Immuno-isolates were probed with anti-caveolin 1, anti-cavin 1, anti-cavin 3, or anti-flotillin 1 antibodies.
(TIF)

Figure S3 **Chemical crosslinking does not perturb the distribution of caveolin 1 or cavins.** (A) Confocal images of cells stably expressing cavin-1-GFP, fixed, and stained with caveolin 1 antibodies after incubation for 1 h in PBS, 1% DMSO (−DSP) or PBS, 1% DMSO, 1.2 mM DSP (+DSP). Bars are 20 µm. (B) Cells treated as in (A), but imaged using total internal reflection microscopy. Bars are 5 µm. (C) Cells stably transfected with either cavin-3-GFP or cavin-3-MiniSOG-mCherry were plated out either separately, or mixed together at a 1∶1 ratio in the same dish. They were cross-linked with DSP, lysed, and immuno-precipiatated with anti-GFP antibodies. The lysates and immuno-precipitates were analysed by Western blotting with the indicated antibodies. Note the absence of cavin-3-MiniSOG-mCherry in all immuno-precipitates.
(TIF)

Figure S4 **Cavin 1, 2, and 3 with a C-terminal TEV-GFP-10×His tag localise correctly and are expressed at low levels.** (A) HeLa cells stably transfected with cavin 1, 2, or 3-TEV-GFP-10×His were aldehyde-fixed, stained with anti-caveolin 1 antibodies, and analysed by TIRF microscopy. (B) Hela cells stably transfected with cavin-3-TEV-GFP-10×His were transfected with plasmid expressing cavin-2-mCherry, fixed, and stained with anti-cavin-1 antibodies, before analysis by confocal microscopy. The lower panels show a zoomed in view of the box in the main panels. Bar is 5 µm. (C) Western blots of lysates from HeLa cells stably transfected with cavin 1, 2, or 3-TEV-GFP-10×His, caveolin-1-GFP, flotillin-2-GFP, or GFP, probed with antibodies against cavin 3 and cavin 1. Note that the expression of endogenous cavin 3 is markedly and specifically reduced in cells stably transfected with cavin-3-TEV-GFP-10×His, and that the expression level of GFP-tagged cavin 3 is comparable to the level of endogenous cavin 3 in the other cell lines. ? indicate nonspecific bands observed using the cavin 3 antibody. These bands are still present in cells lacking the gene for cavin 3 (not shown).
(TIF)

Figure S5 **Cavin 1 co-precipitates cavin 2 and cavin 3 in the absence of caveolin 1, but cavin 2 and cavin 3 do not co-precipitate without cavin 1.** (A) Embryonic fibroblasts from congenic control and *caveolin 1* knockout (KO) mice were transfected with plasmids expressing the constructs denoted by x in each lane (cavin-2-mCh is cavin-2-MiniSOG-mCherry; cavin-3-mCh is cavin-3-MiniSOG-mCherry). Cells were cross-linked with DSP, lysed, and immuno-precipitated with anti-GFP antibodies. The lysates and immunoprecipitates were analysed by Western blotting with the antibodies indicated. * indicates a background band detected by the anti-cavin-3 antibody in cell lysates. (B) Embryonic fibroblasts from congenic control and *cavin 1* knockout (KO) mice were transfected with plasmids expressing cavin-2-GFP and cavin-3-MiniSOG-mCherry (shown as cavin-3-mCh). Cells were cross-linked with DSP, lysed, and immuno-precipitated with anti-GFP antibodies. The lysates and immunoprecipitates were analysed by Western blotting with the antibodies indicated. * indicates a background band detected by the anti-cavin-3 antibody in cell lysates, while<indicates cross-reaction between the anti-cavin-3 antibody or secondary antibody and immunoglobulin heavy chains present in the immunoprecipitates.
(TIF)

Figure S6 **Partial reduction of cross-linked caveolar coat complexes provides evidence for subcomplexes.** (A and B0 HeLa cells stably transfected with caveolin-1-GFP or cavin 1, 2, or 3-GFP were cross-linked with 1.2 mM DSP and lysed in 1% Triton X-100/1% octyl glucoside. The caveolar coat complex was immuno-isolated from each cell lysate using anti-GFP antibodies. Immuno-isolates were incubated with 0, 1, or 2 mM DTT for 15 min at 37°C, boiled for 2 min, and separated by 4–20% SDS-PAGE. Western blots were performed with antibodies against cavin 1 (A) or caveolin 1 (B). (A) Cavin 1 forms a stable trimer. Under nonreducing conditions (no DTT), most cavin 1 is found in oligomeric forms. The cavin 1 trimer (180 kDa), which is stable at 2 mM DTT, is indicated (see also Figure 4D). Note that cavin-1-GFP (indicated by an asterisk) runs slightly above an 80 kDa band of cavin 1. (B) Caveolin 1 forms a stable 350–400 kDa complex, indicated as the caveolin 1 oligomer, and a stable dimer. Note that expression of caveolin-1-GFP alters the molecular weights of oligomeric forms of caveolin 1.
(TIF)

Figure S7 **Sucrose velocity gradients analysing the caveolar coat complex in cells treated with siRNAs to knock down cavin 2, cavin 3, and EHD2 expression.** (A) Cells stably transfected with cavin-1-GFP, cavin-2-GFP, or cavin-3-GFP were transfected with siRNAs to knock down expression of flotillin 1 and 2, cavin 2, or cavin 3. As endogenous cavin 2 cannot be detected with available antibodies in HeLa cells, knock down of the stably expressed cavin-2-GFP provides a way of confirming that the cavin 2 siRNAs function efficiently. Efficiency of knockdown was assessed by Western blotting of cell lysates with the antibodies indicated under each panel. * denotes a background band detected with the anti-cavin-3 antibody: note that this band is not altered by cavin 3 siRNA treatment, while the specific cavin 3 band disappears. (B) Cells stably transfected with cavin-1-GFP were transfected with siRNAs to knock down expression of flotillin 1 and 2, cavin 2, cavin 3, or EHD2 as indicated. Cells were cross-linked with DSP before lysis and analysis of the caveolar coat complex on sucrose gradients as previously. Fractions from the gradients were analysed by Western blotting with the antibodies indicated. * denotes a background band detected with the anti-cavin-3 antibody. Fractions 3–5 (Low Molecular Weight) and 8–10 (High Molecular Weight) were pooled for quantitative side-by-side analysis of total protein levels as shown in Figure 5B and 5C.
(TIF)

Figure S8 **Representative images of caveolae immuno-labelled with anti-GFP, anti-caveolin-1, and anti-cavin-1 antibodies.** HeLa cell lines stably transfected with either cavin 1, 2, or 3-GFP were processed for immuno-electron microscopy, using pre-embedding labeling with anti-GFP primary antibodies, nanogold-conjugated secondary antibodies, and silver enhancement. Untransfected cells were processed in the same way, but were labelled with anti-caveolin-1 or anti-cavin-1 antibodies as shown.
(TIF)

Figure S9 **Cavin 1, 2, and 3 with a C-terminal MiniSOG-mCherry tag localise correctly.** (A) HeLa cells transfected with cavin 1, 2, or 3-MiniSOG-mCherry were aldehyde-fixed, stained with anti-caveolin-1 antibodies, and analyzed by TIRF microscopy. (B) RPE cells stably transfected with cavin 1, 2, or 3-MiniSOG-mCherry were aldehyde-fixed and inspected by confocal microscopy. Note the pronounced polarization of cavins at the cell rear.
(TIF)

Figure S10 **Cavin-MiniSOG proteins specifically label caveolar membranes.** Transmission electron micrograph of a photooxidized and osmium-tetraoxide stained RPE cell stably transfected with cavin-2-MiniSOG-mCherry. Boxed regions 1 and 2 show caveolar membranes strongly labeled with an electron-dense stain. Boxed region 3 shows small vesicles, apparently clathrin-coated, which are not stained with an electron-dense deposit. Note that mitochondria and putative endosomal membranes (asterisks) are not labeled.
(TIF)

File S1 **Excel file containing peptides detected by mass spectrometry of caveolin-1-GFP immunoprecipites.**
(XLSX)

Movie S1 **3D electron tomography of the caveolar coat visualised with cavin-3-miniSOG.** A sequence of z slices through a 150–200 nm section is shown. The tomogram was recorded at 150 kV at 18k with a pixel size of 0.5 nm. Images are bin 2 back-projections.
(MOV)

Movie S2 **3D electron tomography of the caveolar coat visualised with cavin-3-miniSOG.** Shown is a maximum intensity projection of a subregion shown in Movie S1.
(MOV)

Table S1 **Pearson correlation coefficients describing co-localisation between cavin-GFP or cavin-MiniSOG constructs and caveolin 1.** Also included are comparisons of cavin-1-GFP and flotillin 1, where there is no co-localisation visible by eye, and cavin-1-GFP and cavin 1 antibody staining, where there should be maximal co-localisation.
(DOCX)

## Abbreviations

EM: electron microscopy
GFP: green fluorescent protein
MEF: mouse embryonic fibroblast
MiniSOG: Mini Singlet Oxygen Generator
PAGE: poly-acrylamide gel electrophoresis
