# Supplementary figures and images for: Molecular Composition and Ultrastructure of the Caveolar Coat Complex
Source: PLoS Biol. 2013 Aug 27;11(8):e1001640. doi: 10.1371/journal.pbio.1001640 (PMC3754886; doi:10.1371/journal.pbio.1001640)

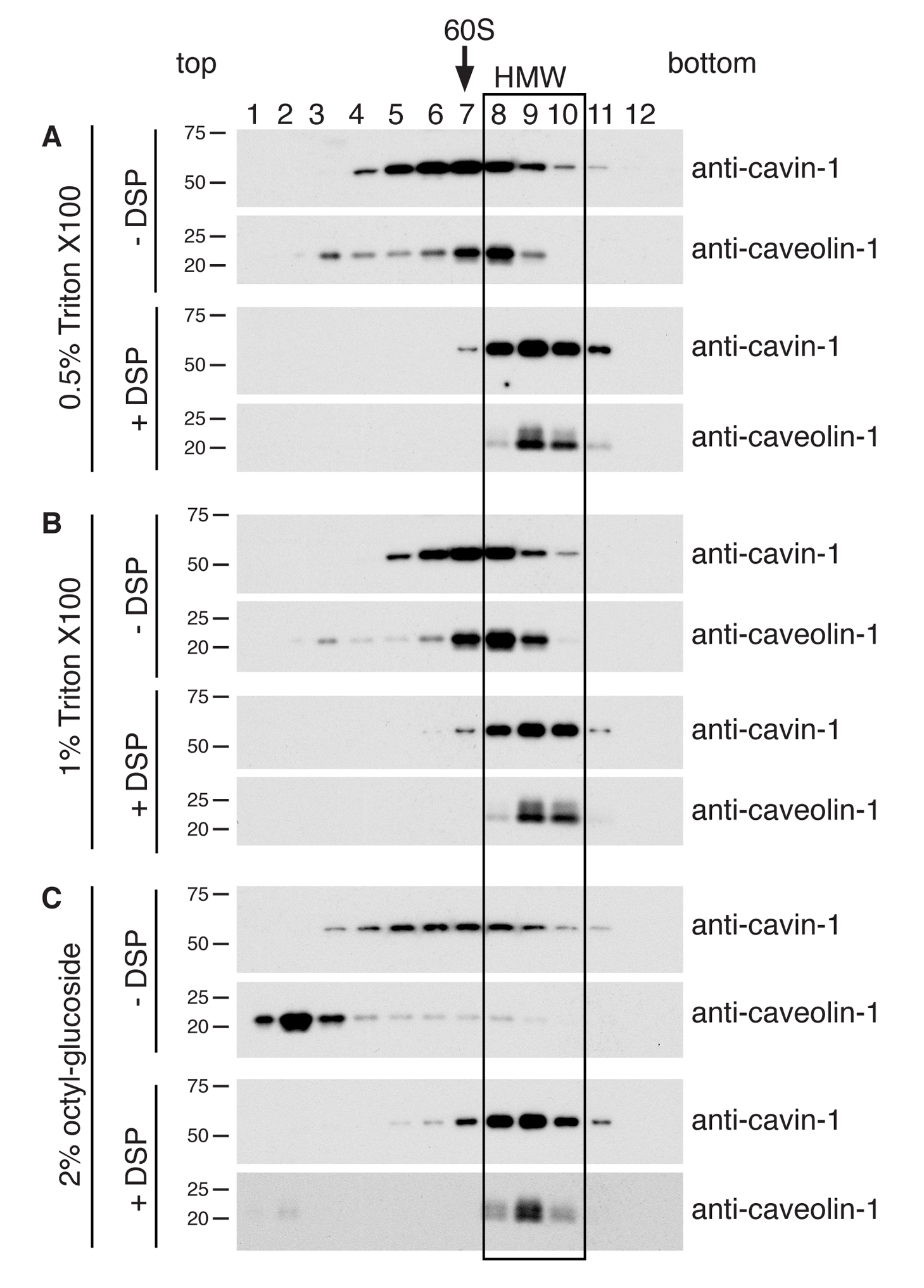

Supplement: Figure S1 — Cavin/caveolin complexes are sensitive to detergent, but can be stabilized by crosslinking. HeLa cells, either cross-linked with DSP or left untreated, were solubilised in either 0.5% Triton X-100 (A), 1% Triton X-100 (B), or 2% octyl glucoside (C). Lysates were fractionated on 10–40% sucrose gradients, followed by Western blotting of gradient fractions 1–12 using antibodies against caveolin 1 or cavin 1. Without cross-linking, oligomeric complexes of caveolin 1 are sensitive to the detergent used for solubilisation. Full dissociation of caveolin oligomers was achieved with 2% octyl glucoside (C top) or a combination of 1% Triton X-100/1% octyl glucoside (Figure 1A). Note that cross-linking stabilises a high molecular weight (HMW) complex of both caveolin 1 and cavin 1 in all detergents tested. (TIF) [file pbio.1001640.s001.tif]

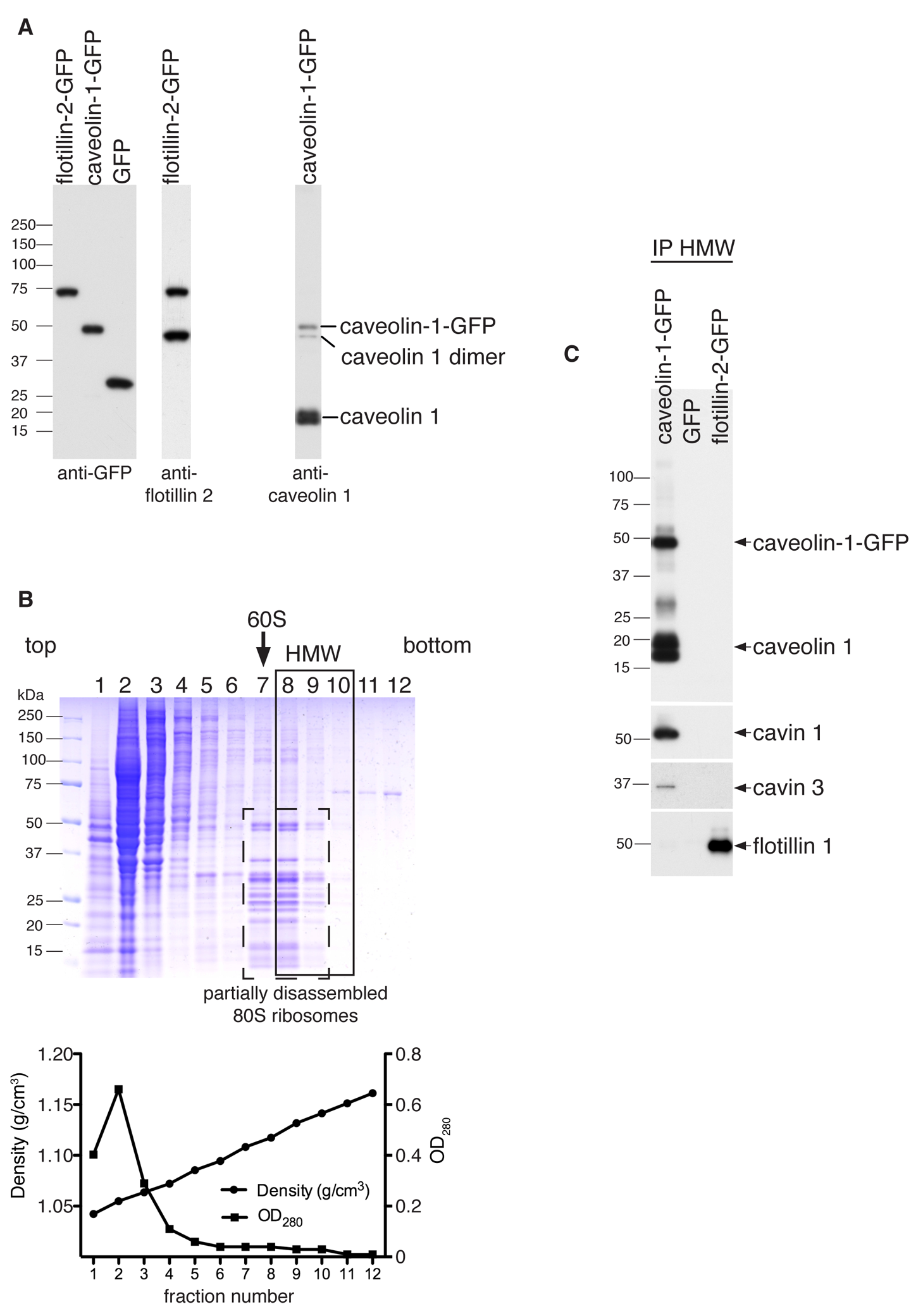

Supplement: Figure S2 — Isolation of the caveolar coat complex using HeLa cells stably transfected with caveolin-1-GFP. (A) Western blots of lysates from HeLa cells stably transfected with caveolin-1-GFP, flotillin-2-GFP, or GFP. Membranes were probed with anti-GFP, anti-flotillin 2, or anti-caveolin 1 antibodies. Note that caveolin-1-GFP is expressed at low levels relative to endogenous caveolin 1, and that there is no detectable proteolysis of caveolin-1-GFP. (B) Coomassie-stained protein gel of gradient fractions 1–12 prepared from DSP-cross-linked HeLa cells. The HMW peak of fractions 8–10 that contain caveolin 1 and cavin 1 is boxed. Fractions 7–9 are rich in ribosomal proteins (dashed box). The 60S peak obtained for purified 60S ribosomal subunit is indicated. (C) HeLa cells stably transfected with caveolin-1-GFP, flotillin-2-GFP, or GFP were cross-linked with DSP and lysed in 1% Triton X-100/1% octyl glucoside. Lysates were fractionated on 10–40% sucrose gradients and pooled HMW fractions 8–10 used for immuno-isolation of the caveolar coat complex. Immuno-isolates were probed with anti-caveolin 1, anti-cavin 1, anti-cavin 3, or anti-flotillin 1 antibodies. (TIF) [file pbio.1001640.s002.tif]

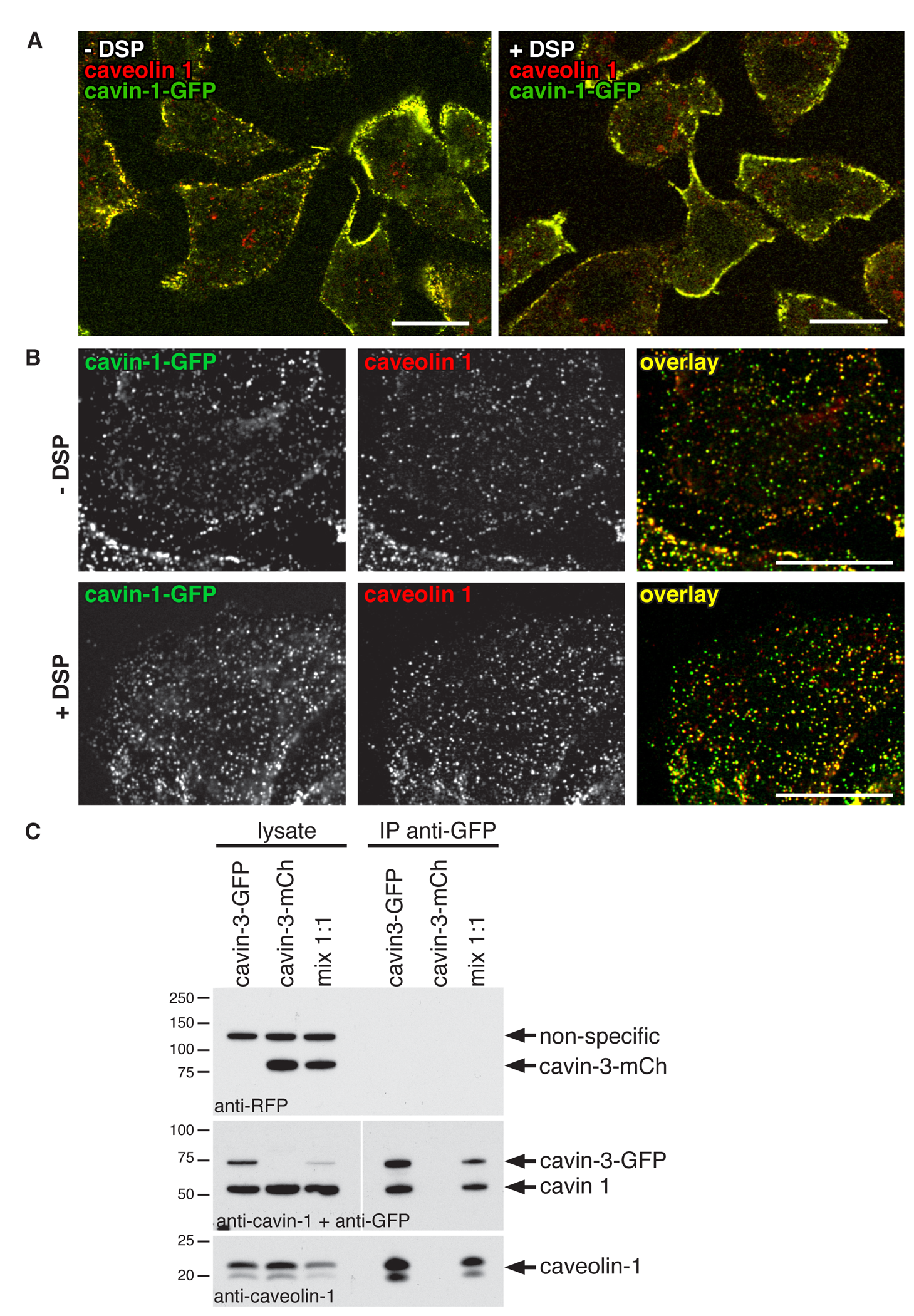

Supplement: Figure S3 — Chemical crosslinking does not perturb the distribution of caveolin 1 or cavins. (A) Confocal images of cells stably expressing cavin-1-GFP, fixed, and stained with caveolin 1 antibodies after incubation for 1 h in PBS, 1% DMSO (−DSP) or PBS, 1% DMSO, 1.2 mM DSP (+DSP). Bars are 20 µm. (B) Cells treated as in (A), but imaged using total internal reflection microscopy. Bars are 5 µm. (C) Cells stably transfected with either cavin-3-GFP or cavin-3-MiniSOG-mCherry were plated out either separately, or mixed together at a 1∶1 ratio in the same dish. They were cross-linked with DSP, lysed, and immuno-precipiatated with anti-GFP antibodies. The lysates and immuno-precipitates were analysed by Western blotting with the indicated antibodies. Note the absence of cavin-3-MiniSOG-mCherry in all immuno-precipitates. (TIF) [file pbio.1001640.s003.tif]

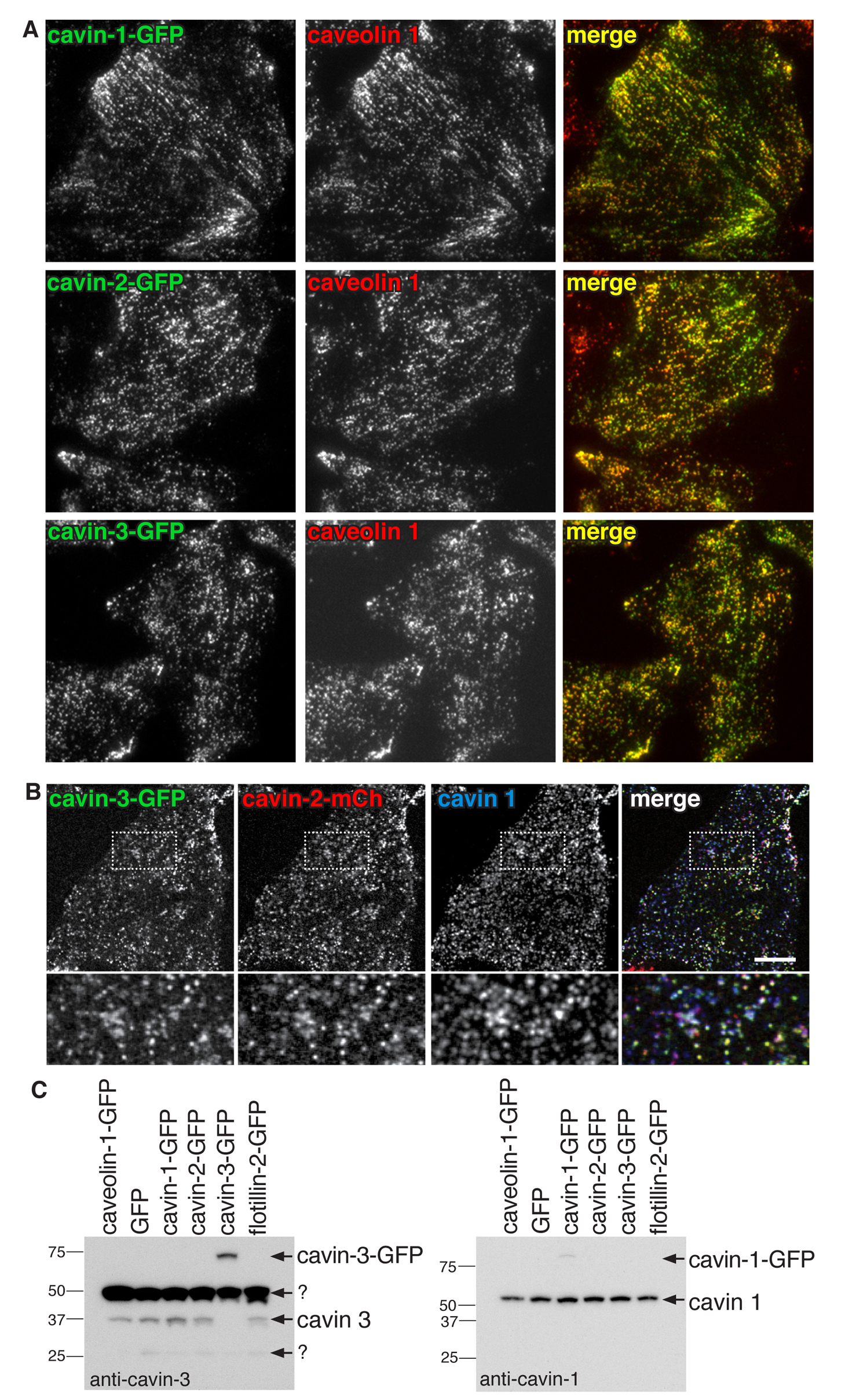

Supplement: Figure S4 — Cavin 1, 2, and 3 with a C-terminal TEV-GFP-10×His tag localise correctly and are expressed at low levels. (A) HeLa cells stably transfected with cavin 1, 2, or 3-TEV-GFP-10×His were aldehyde-fixed, stained with anti-caveolin 1 antibodies, and analysed by TIRF microscopy. (B) Hela cells stably transfected with cavin-3-TEV-GFP-10×His were transfected with plasmid expressing cavin-2-mCherry, fixed, and stained with anti-cavin-1 antibodies, before analysis by confocal microscopy. The lower panels show a zoomed in view of the box in the main panels. Bar is 5 µm. (C) Western blots of lysates from HeLa cells stably transfected with cavin 1, 2, or 3-TEV-GFP-10×His, caveolin-1-GFP, flotillin-2-GFP, or GFP, probed with antibodies against cavin 3 and cavin 1. Note that the expression of endogenous cavin 3 is markedly and specifically reduced in cells stably transfected with cavin-3-TEV-GFP-10×His, and that the expression level of GFP-tagged cavin 3 is comparable to the level of endogenous cavin 3 in the other cell lines. ? indicate nonspecific bands observed using the cavin 3 antibody. These bands are still present in cells lacking the gene for cavin 3 (not shown). (TIF) [file pbio.1001640.s004.tif]

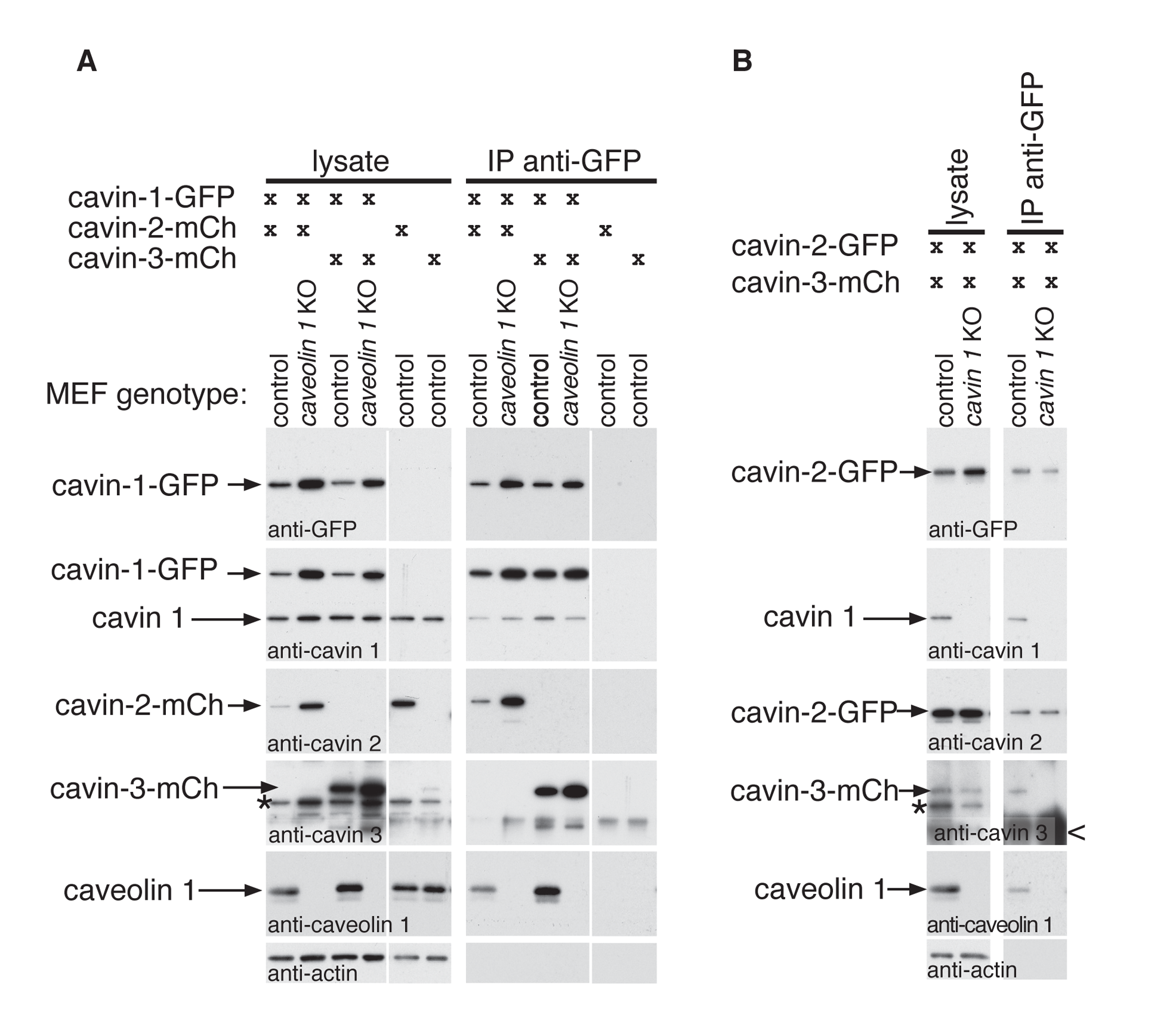

Supplement: Figure S5 — Cavin 1 co-precipitates cavin 2 and cavin 3 in the absence of caveolin 1, but cavin 2 and cavin 3 do not co-precipitate without cavin 1. (A) Embryonic fibroblasts from congenic control and caveolin 1 knockout (KO) mice were transfected with plasmids expressing the constructs denoted by x in each lane (cavin-2-mCh is cavin-2-MiniSOG-mCherry; cavin-3-mCh is cavin-3-MiniSOG-mCherry). Cells were cross-linked with DSP, lysed, and immuno-precipitated with anti-GFP antibodies. The lysates and immunoprecipitates were analysed by Western blotting with the antibodies indicated. * indicates a background band detected by the anti-cavin-3 antibody in cell lysates. (B) Embryonic fibroblasts from congenic control and cavin 1 knockout (KO) mice were transfected with plasmids expressing cavin-2-GFP and cavin-3-MiniSOG-mCherry (shown as cavin-3-mCh). Cells were cross-linked with DSP, lysed, and immuno-precipitated with anti-GFP antibodies. The lysates and immunoprecipitates were analysed by Western blotting with the antibodies indicated. * indicates a background band detected by the anti-cavin-3 antibody in cell lysates, while<indicates cross-reaction between the anti-cavin-3 antibody or secondary antibody and immunoglobulin heavy chains present in the immunoprecipitates. (TIF) [file pbio.1001640.s005.tif]

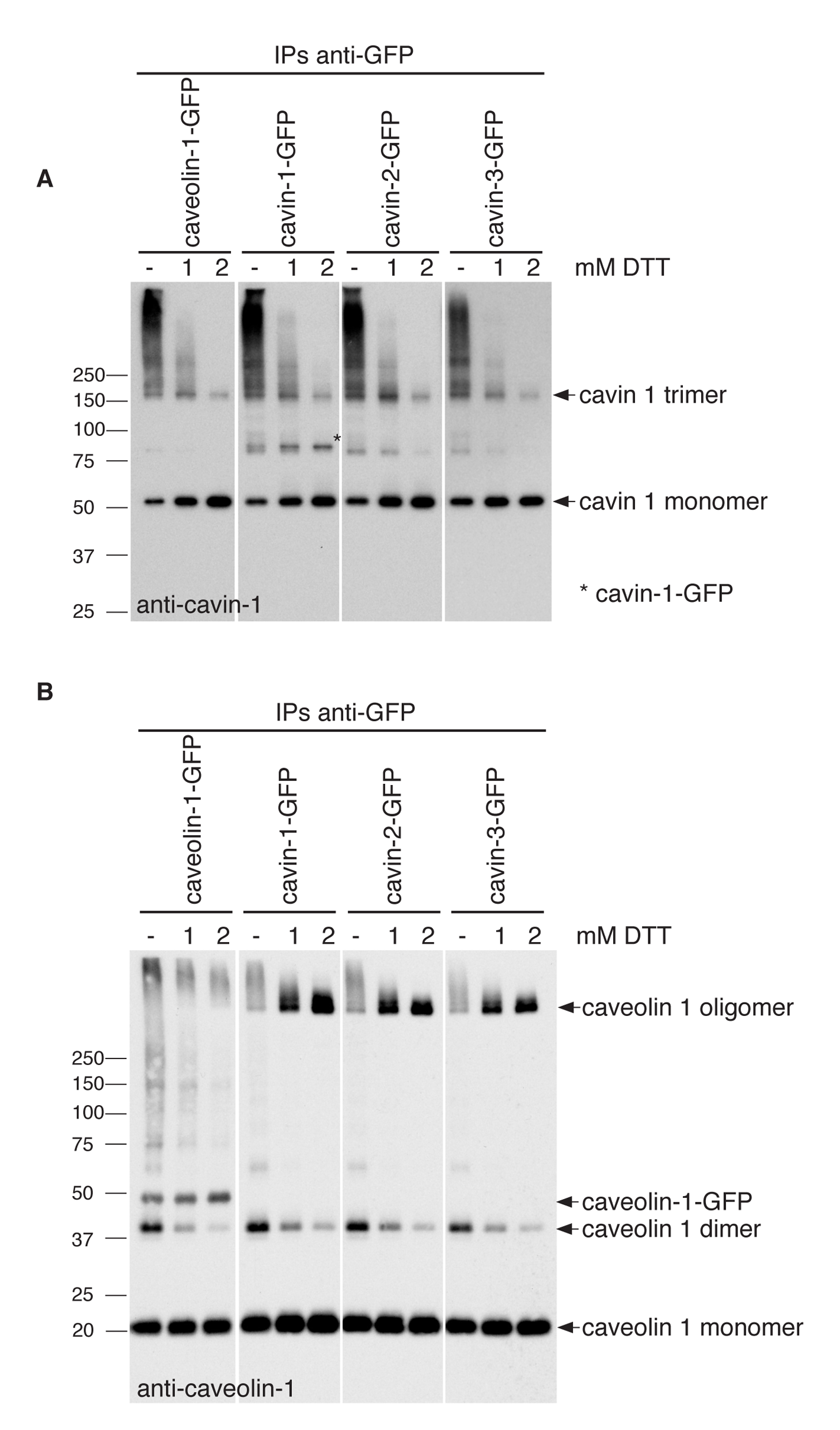

Supplement: Figure S6 — Partial reduction of cross-linked caveolar coat complexes provides evidence for subcomplexes. (A and B0 HeLa cells stably transfected with caveolin-1-GFP or cavin 1, 2, or 3-GFP were cross-linked with 1.2 mM DSP and lysed in 1% Triton X-100/1% octyl glucoside. The caveolar coat complex was immuno-isolated from each cell lysate using anti-GFP antibodies. Immuno-isolates were incubated with 0, 1, or 2 mM DTT for 15 min at 37°C, boiled for 2 min, and separated by 4–20% SDS-PAGE. Western blots were performed with antibodies against cavin 1 (A) or caveolin 1 (B). (A) Cavin 1 forms a stable trimer. Under nonreducing conditions (no DTT), most cavin 1 is found in oligomeric forms. The cavin 1 trimer (180 kDa), which is stable at 2 mM DTT, is indicated (see also Figure 4D). Note that cavin-1-GFP (indicated by an asterisk) runs slightly above an 80 kDa band of cavin 1. (B) Caveolin 1 forms a stable 350–400 kDa complex, indicated as the caveolin 1 oligomer, and a stable dimer. Note that expression of caveolin-1-GFP alters the molecular weights of oligomeric forms of caveolin 1. (TIF) [file pbio.1001640.s006.tif]

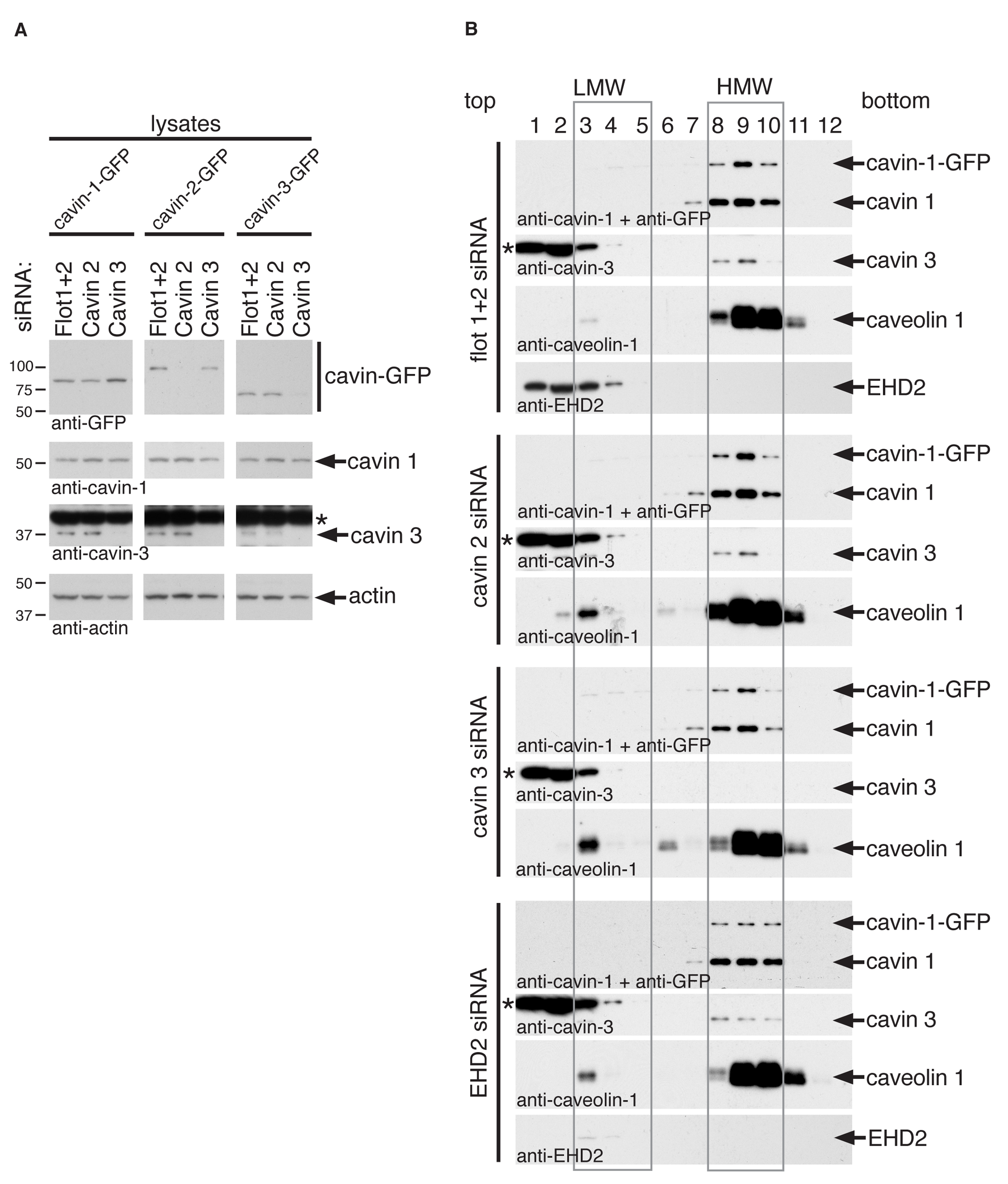

Supplement: Figure S7 — Sucrose velocity gradients analysing the caveolar coat complex in cells treated with siRNAs to knock down cavin 2, cavin 3, and EHD2 expression. (A) Cells stably transfected with cavin-1-GFP, cavin-2-GFP, or cavin-3-GFP were transfected with siRNAs to knock down expression of flotillin 1 and 2, cavin 2, or cavin 3. As endogenous cavin 2 cannot be detected with available antibodies in HeLa cells, knock down of the stably expressed cavin-2-GFP provides a way of confirming that the cavin 2 siRNAs function efficiently. Efficiency of knockdown was assessed by Western blotting of cell lysates with the antibodies indicated under each panel. * denotes a background band detected with the anti-cavin-3 antibody: note that this band is not altered by cavin 3 siRNA treatment, while the specific cavin 3 band disappears. (B) Cells stably transfected with cavin-1-GFP were transfected with siRNAs to knock down expression of flotillin 1 and 2, cavin 2, cavin 3, or EHD2 as indicated. Cells were cross-linked with DSP before lysis and analysis of the caveolar coat complex on sucrose gradients as previously. Fractions from the gradients were analysed by Western blotting with the antibodies indicated. * denotes a background band detected with the anti-cavin-3 antibody. Fractions 3–5 (Low Molecular Weight) and 8–10 (High Molecular Weight) were pooled for quantitative side-by-side analysis of total protein levels as shown in Figure 5B and 5C. (TIF) [file pbio.1001640.s007.tif]

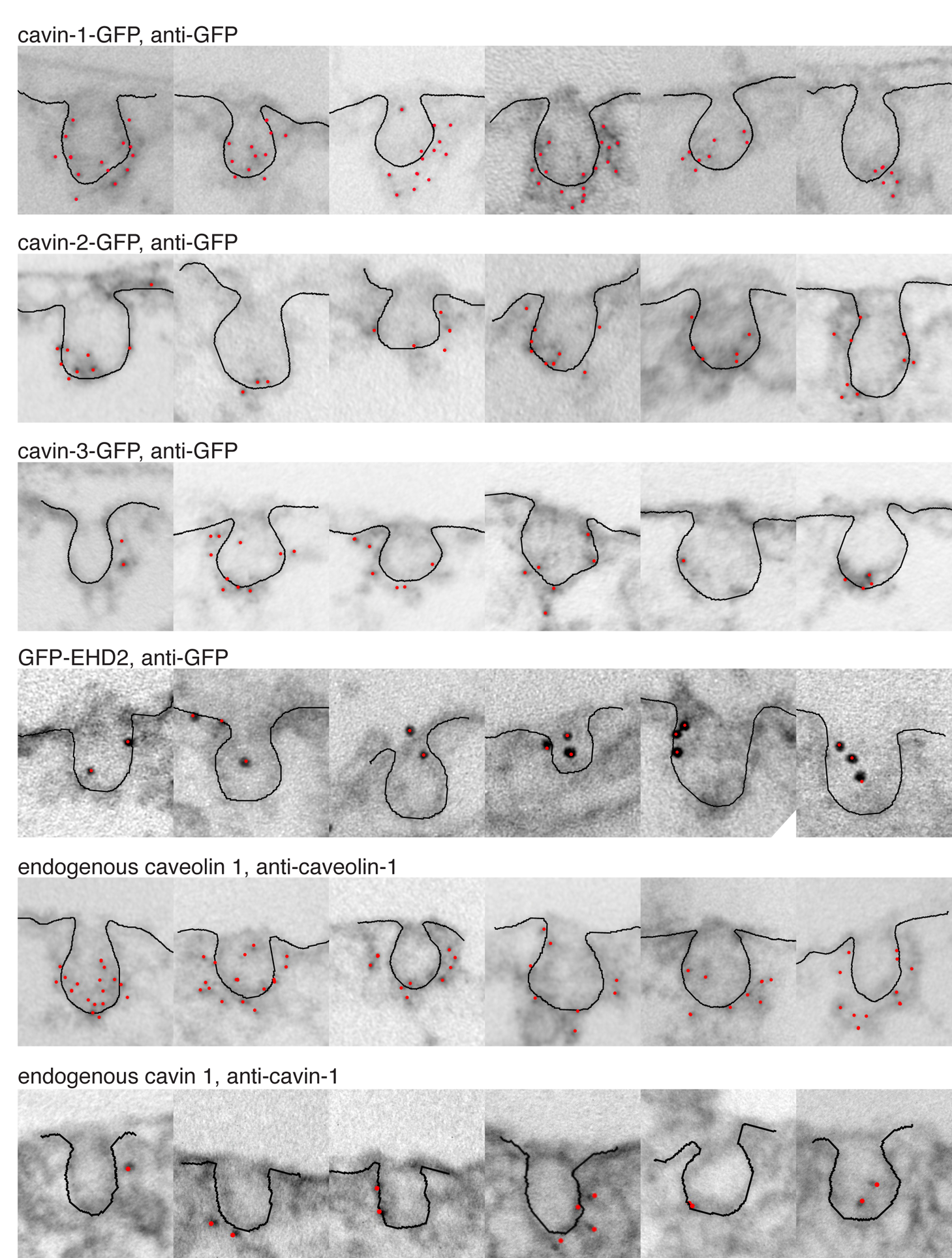

Supplement: Figure S8 — Representative images of caveolae immuno-labelled with anti-GFP, anti-caveolin-1, and anti-cavin-1 antibodies. HeLa cell lines stably transfected with either cavin 1, 2, or 3-GFP were processed for immuno-electron microscopy, using pre-embedding labeling with anti-GFP primary antibodies, nanogold-conjugated secondary antibodies, and silver enhancement. Untransfected cells were processed in the same way, but were labelled with anti-caveolin-1 or anti-cavin-1 antibodies as shown. (TIF) [file pbio.1001640.s008.tif]

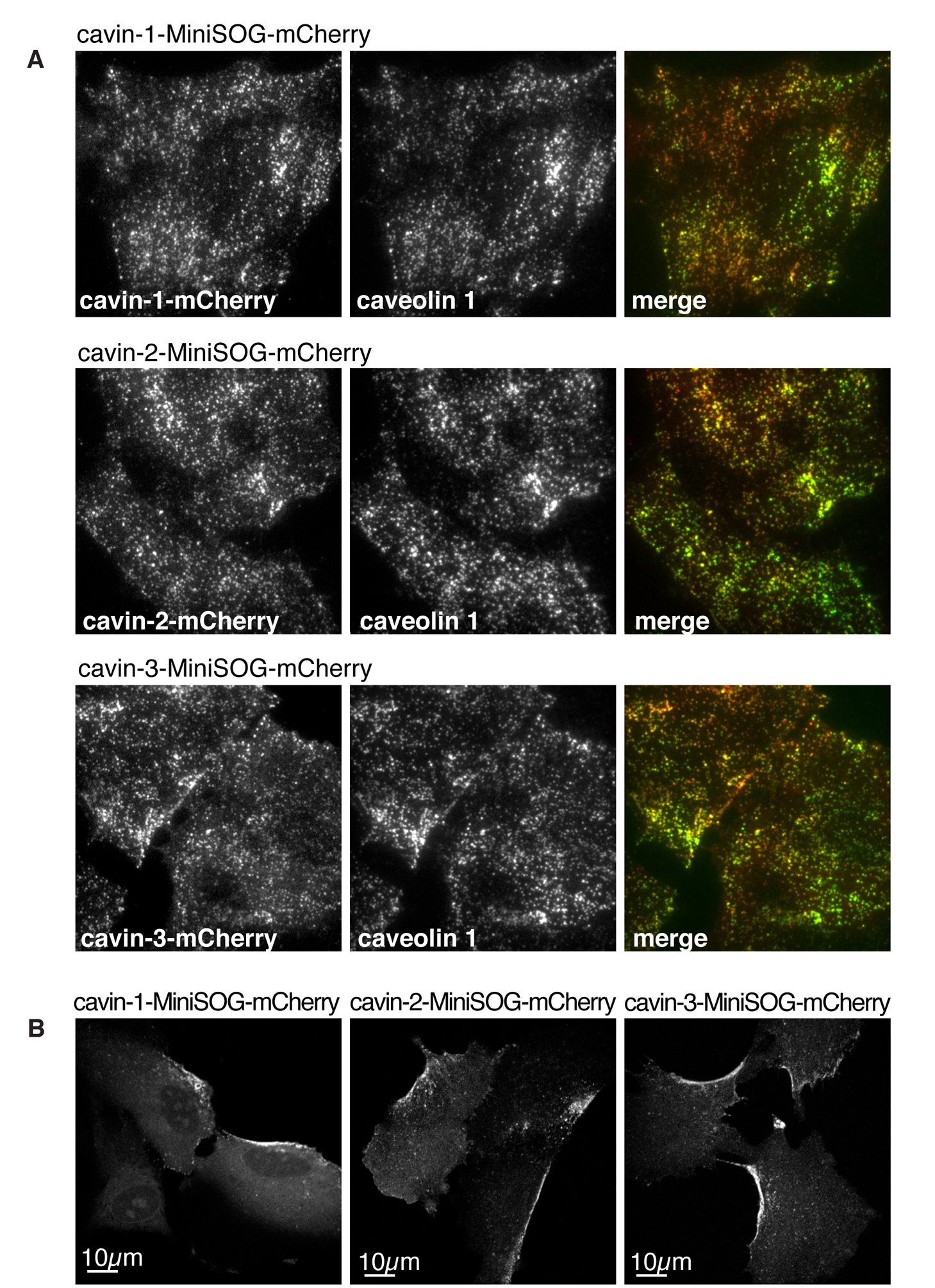

Supplement: Figure S9 — Cavin 1, 2, and 3 with a C-terminal MiniSOG-mCherry tag localise correctly. (A) HeLa cells transfected with cavin 1, 2, or 3-MiniSOG-mCherry were aldehyde-fixed, stained with anti-caveolin-1 antibodies, and analyzed by TIRF microscopy. (B) RPE cells stably transfected with cavin 1, 2, or 3-MiniSOG-mCherry were aldehyde-fixed and inspected by confocal microscopy. Note the pronounced polarization of cavins at the cell rear. (TIF) [file pbio.1001640.s009.tif]

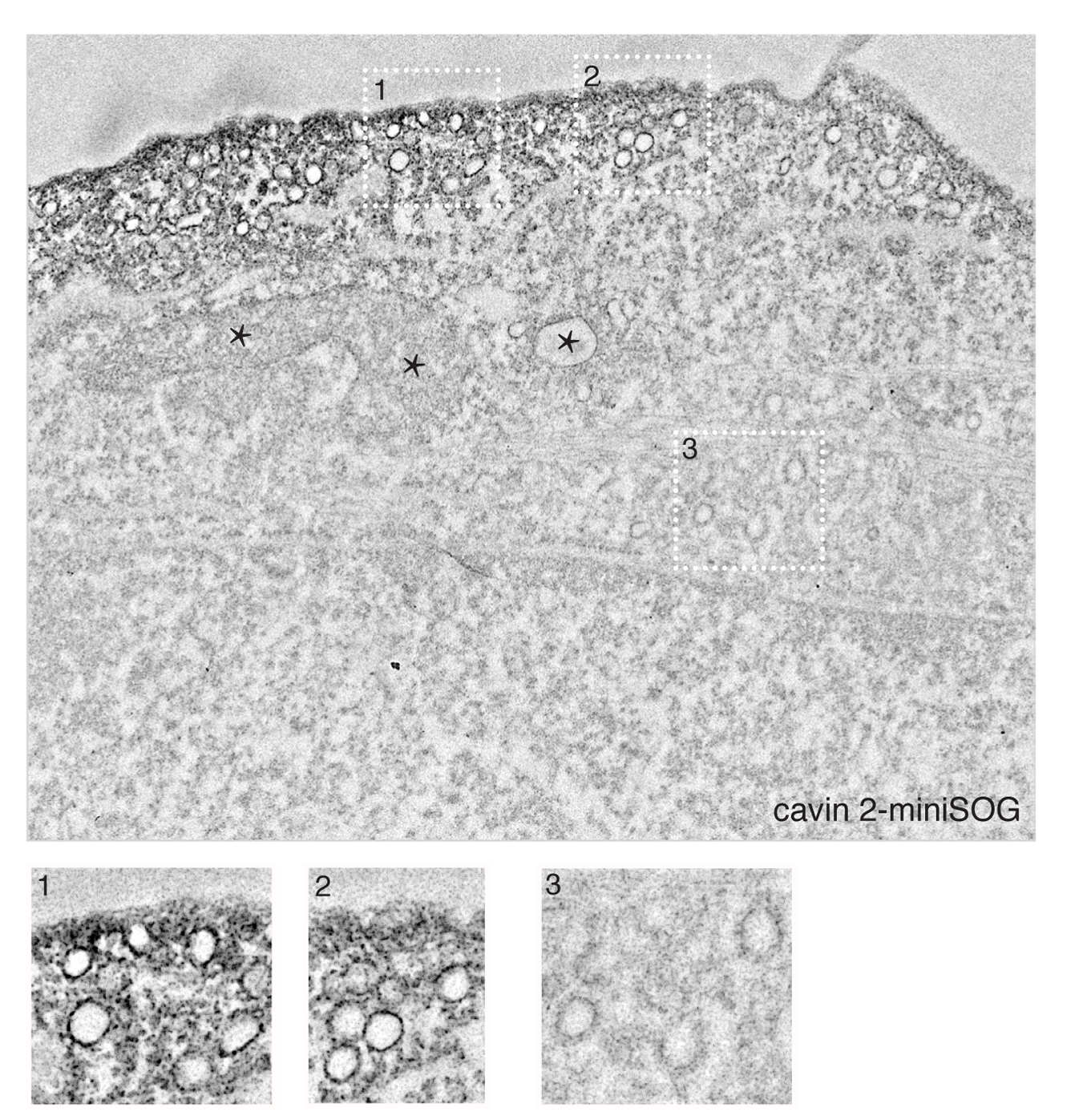

Supplement: Figure S10 — Cavin-MiniSOG proteins specifically label caveolar membranes. Transmission electron micrograph of a photooxidized and osmium-tetraoxide stained RPE cell stably transfected with cavin-2-MiniSOG-mCherry. Boxed regions 1 and 2 show caveolar membranes strongly labeled with an electron-dense stain. Boxed region 3 shows small vesicles, apparently clathrin-coated, which are not stained with an electron-dense deposit. Note that mitochondria and putative endosomal membranes (asterisks) are not labeled. (TIF) [file pbio.1001640.s010.tif]
