# Supplementary material for: Molecular Composition and Ultrastructure of the Caveolar Coat Complex
Source: PLoS Biol. 2013 Aug 27;11(8):e1001640. doi: 10.1371/journal.pbio.1001640 (PMC3754886; doi:10.1371/journal.pbio.1001640)
Supplement: Table S1 — Pearson correlation coefficients describing co-localisation between cavin-GFP or cavin-MiniSOG constructs and caveolin 1. Also included are comparisons of cavin-1-GFP and flotillin 1, where there is no co-localisation visible by eye, and cavin-1-GFP and cavin 1 antibody staining, where there should be maximal co-localisation. (DOCX) [file pbio.1001640.s014.docx]

|  | Pearson correlation coefficient, mean | N | SD |
| --- | --- | --- | --- |
| **cavin-1-GFP and caveolin 1 antibody** | **0.71** | 12 | 0.21 |
| **cavin-2-GFP and caveolin 1 antibody** | **0.74** | 11 | 0.32 |
| **cavin-3-GFP and caveolin 1 antibody** | **0.69** | 10 | 0.14 |
| **cavin-1-GFP and cavin 1 antibody** | **0.78** | 11 | 0.09 |
| **cavin-1-GFP and flotillin 1 antibody** | **-0.04** | 8 | 0.09 |
| **cavin-1-MiniSOG and caveolin 1 antibody** | **0.65** | 12 | 0.12 |
| **cavin-2-MiniSOG and caveolin 1 antibody** | **0.78** | 13 | 0.21 |
| **cavin-3-MiniSOG and caveolin 1 antibody** | **0.60** | 8 | 0.11 |

**Supplementary Table 1**

**Pearson correlation coefficients describing co-localisation between cavin-GFP or cavin-MiniSOG constructs and caveolin 1.** Also included are comparisons of cavin-1-GFP and flotillin 1, where there is no co-localisation visible by eye, and cavin-1-GFP and cavin 1 antibody staining, where there should be maximal co-localisation
